# Impact of COVID-19 forecast visualizations on pandemic risk perceptions

**DOI:** 10.1038/s41598-022-05353-1

**Published:** 2022-02-07

**Authors:** Lace Padilla, Helia Hosseinpour, Racquel Fygenson, Jennifer Howell, Rumi Chunara, Enrico Bertini

**Affiliations:** 1 Cognitive and Information Sciences Department, University of California Merced, Merced, 95340 USA; 2 Computer Science and Engineering Department, New York University, New York, 748766 USA; 3 Psychological Sciences Department, University of California Merced, Merced, 95340 USA; 4 Present Address: Computer Science and Engineering Department, Northeastern University, Boston, 02115 USA

**Keywords:** Psychology, Human behaviour, Scientific data, Computer science

## Abstract

People worldwide use SARS-CoV-2 (COVID-19) visualizations to make life and death decisions about pandemic risks. Understanding how these visualizations influence risk perceptions to improve pandemic communication is crucial. To examine how COVID-19 visualizations influence risk perception, we conducted two experiments online in October and December of 2020 (*N* = 2549) where we presented participants with 34 visualization techniques (available at the time of publication on the CDC’s website) of the same COVID-19 mortality data. We found that visualizing data using a cumulative scale consistently led to participants believing that they and others were at more risk than before viewing the visualizations. In contrast, visualizing the same data with a weekly incident scale led to variable changes in risk perceptions. Further, uncertainty forecast visualizations also affected risk perceptions, with visualizations showing six or more models increasing risk estimates more than the others tested. Differences between COVID-19 visualizations of the same data produce different risk perceptions, fundamentally changing viewers’ interpretation of information.

## Introduction

The fast-acting and deadly nature of SARS-CoV-2 (also known as COVID-19 and the coronavirus) have prompted scientists and media outlets to produce thousands of visualizations to convey the pandemic risk^1^. Government organizations and the public rely heavily on line charts of COVID-19 infection and morbidity rates to make decisions that have life-or-death consequences for the health of the global population^2^. Many line charts of COVID-19 data also include forecasts using a wide range of modeling techniques to show predicted COVID-19 trends. All forecasts inherently include *uncertainty*, and there are multiple ways to visualize the uncertainty associated with COVID-19 forecasts. Although different fields have various definitions of uncertainty (for a discussion, see Ref.^3^), we focus on quantified forms of error and variability in pandemic forecasts, such as those communicated through confidence intervals^4^.

Understanding the effects of forecast visualizations on risk perceptions during a pandemic is vital for ensuring that the public takes appropriate actions to mitigate the spread of a virus and reduce personal risk. Because we have numerous ways to visualize the same data, risk communicators may inadvertently use a visualization technique that minimizes viewers’ perception of their pandemic risk, indirectly contributing to inappropriate actions. This work aims to determine if COVID-19 forecast visualizations impact pandemic risk perception. Further, we seek to test if standard uncertainty visualization techniques produce differences in people’s beliefs about the pandemic risks to themselves and others.

Effectively designed visualizations can be powerful tools for communicating health risks, particularly those that include probability, which can be highly challenging for many people to understand. For example, one study found that approximately 18% of college-educated people incorrectly answer the question, “Which represents the larger risk: 1%, 5%, or 10%?”^5^. Rather than requiring the completion of mathematical calculations, visualizations allow viewers to extract patterns in the data intuitively. Within the context of health care, research finds that effectively designed visual aids can improve risk assessments, reduce biases, increase healthy behaviors, and support trust in treatments (for reviews, see^6–8^). Similarly, work outside the health care context finds that poorly designed visualizations can mislead and confuse. For example, truncating the y-axis can result in viewers overestimating minor differences^9, 10^, rainbow color schemes can create visual artifacts that viewers think are properties of the data^11, 12^, and hurricane forecasts that use a cone to show the path of the storm lead people to incorrectly think that the storm is growing in size^13^. Both the positive and negative effects of visualizations illustrate the strong hold that visualizations have on our understanding of data.

**Figure 1:**
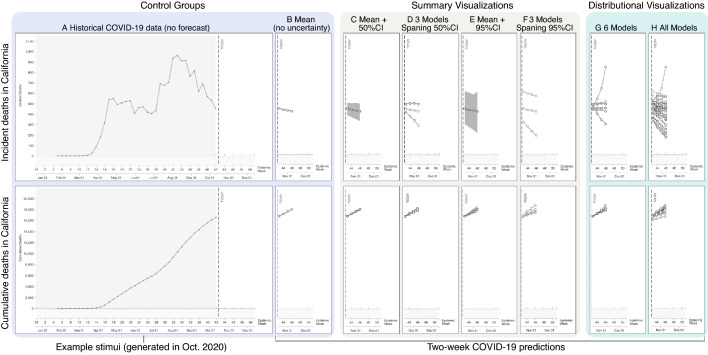
Leftmost panels show line charts displaying historical COVID-19 mortality data with incident (top) and cumulative (bottom) y-axes used in Experiment 1. The remaining panels show the uncertainty visualization forecasts that were added to the historical data. Full stimuli sets are available in the Supplementary Information. Eight visualization techniques (columns of the figure) and two axes (rows of the figure) were tested for a total of 16 stimuli types. The stimuli were generated using COVID-19 ForecastHub created by the Reich Lab from the University of Massachusetts Amherst. The CDC used these line charts at the time of publication^14^.

Risk communicators have responded to the urgent need to inform the public about COVID-19 risks with an unprecedented deployment of data visualizations (for a review of over 600 COVID-19 visualizations, see Ref.^1^), without evidence of how these visualizations influence the global population’s understanding of pandemic risk. Of the visualizations developed by government agencies, news media, and academics, the most common is a line chart that shows COVID-19 metrics over time (e.g., time-series data)^1^. For example, Fig. 1A (leftmost column) shows two COVID-19 line charts from the Reich Lab COVID-19 Forecast Hub, a central repository of forecasts and predictions from over 50 international research groups. The Centers for Disease Control and Prevention (CDC) features the Reich Lab’s charts on the CDC’s website at the time of publication^14^. Both charts use the same data and show time on the x-axis. Figure 1A (top) uses a linear y-axis that shows weekly COVID-19 death counts in California (i.e., *incident* y-axis). Incident y-axis scales can show upward or downward trends based on increasing or decreasing cases compared to prior weeks. In contrast, Fig. 1A (bottom) shows the same data but displayed with a linear *cumulative* y-axis that summarizes the deaths in California. Incident y-axis scales can show variable trends, whereas cumulative scales can show only an upward or flat trend of the same data. Risk communicators have no guidance about when to use each scale or why one scale would be preferable.

Line charts became popular over a century ago to intuitively visualize time-series data^15^. Recent research on COVID-19-specific line charts has examined viewers’ understanding of linear and logarithmic y-axes, finding that viewers who read a graph with a linear y-axis answer comprehension questions more accurately, predict the data’s future trends more reasonably, and report being more worried about the COVID-19 health crisis^16^. Outside the COVID-19-specific literature, visualization research suggests that the audience’s interpretation of line charts is influenced by truncating y-axes and varying the scaling of the axes^9, 17, 18^. Together, these studies suggest that laypeople’s interpretation of line charts and their consequent perception of personal risk is meaningfully impacted by the scaling of the y-axis.

Many COVID-19 line charts also include forecasts of the COVID-19 trends in the coming weeks. A survey of COVID-19 visualizations found that 84% of forecast visualizations include the prediction’s uncertainty^1^. A growing body of research offers mixed findings regarding the efficacy of visualizing uncertainty. Multiple studies have found that conventional uncertainty visualizations, such as 95% confidence intervals, can be confusing or lead to misinterpretations of data^13, 19–24^, even for experts^23, 25^, and when controlling for education^26^. Widespread misconceptions about the meaning and interpretations of standard statistical concepts related to uncertainty (e.g., frequentist confidence intervals, Bayesian creditable intervals, and variability^27, 28^) form the foundation of misunderstandings of uncertainty visualizations. Poorly designed visualizations can add complexity and visual-spatial biases^29^ to statistical concepts that people already struggle to understand. For example, the Cone of Uncertainty, produced by the National Hurricane Center, shows a 66% confidence interval around the storm’s predicted path. It starts as a point—the hurricane’s current position—and widens to cover areas that the storm has a 66% probability of crossing in the following five days. The resultant visualization looks like a semitransparent cone with a line down the middle overlaid on a map. Research demonstrates that people misinterpret the increasing cone size as the storm’s size and intensity growing over time^13^. This misinterpretation persists even after training on interpreting the forecast correctly^30^ (see also^19^). Indeed, both novices^13, 20–24, 26^ and published researchers in psychology, neuroscience, and medicine misinterpret confidence intervals^25^ (for reviews of errors in uncertainty visualization, see^20, 31^). Of the 48 COVID-19 forecast line charts reviewed by Zhang et al.^1^, 60% used confidence intervals to convey uncertainty (see Fig. 1C,E for examples). The CDC used a 95% confidence interval as their default visualization to convey COVID-19 forecast predictions when this work was conducted.

Given that confidence intervals can produce biases, some researchers have advocated for text to convey uncertainty rather than visualizations^20^. Zhang et al.^1^ found that 10% of COVID-19 visualizations used text to convey uncertainty. Skeptics of uncertainty visualizations cite biases that visualizations create, studies that do not find differences between uncertainty intervals and textual expressions of uncertainty (e.g.,^32, 33^), and differences that diminish when participants have a longer time to complete a task^34^. Visual intervals and text *summarize* continuous probabilistic data into discrete values such as mean, mode, and 95% confidence intervals (see, Fig. 1C–F for examples). Summaries of probabilistic data, visual or textual, are discrete, and viewers interpret these expressions of data discretely (i.e., dichotomous thinking). However, studies find that, compared to textual expressions, well-designed *distributional* uncertainty visualizations (see Fig. 1G,H for examples) produce better accuracy^35, 36^ and require less mental effort to understand^35^. A parallel body of work finds that distributional uncertainty visualizations evoke a less biased^13, 37^ and more accurate understanding of the data^21, 24, 36, 38^ than summaries (e.g., mean, 95% confidence and containment intervals) of the same data, while requiring less mental effort^35^. For instance, an *ensemble* visualization^39^ that uses lines to show a distribution of routes the hurricane could take eliminates the biases produced by the Cone of Uncertainty^13, 37^ (for examples with COVID-19 data, see Fig. 1G,H). Zhang et al.^1^ found that 29% of COVID-19 visualizations expressed uncertainty using multiple models or scenarios.

## Results

To test if pandemic visualizations impact people’s risk perceptions, we conducted two online experiments in October (Experiment 1, *N* = 1200) and December (Experiment 2,  *N* = 1350) 2020, during the height of the COVID-19 pandemic in the United States. These studies examine the impact of real-time uncertainty visualizations on participants’ understanding of their risks during a pandemic. For the primary outcome measure, participants responded to a series of 12 questions using 7-point Likert scales where higher numbers indicated greater risk (see “Materials and methods” section for full question text and scales). Participants viewed one of the 16 COVID-19 data visualizations (shown in Fig. 1) along with brief instructions about the different elements in the chart.

We selected the stimuli in Experiment 1 based on modern theories of uncertainty communication, which make distinctions between summary (e.g., Fig. 1C–F) and distributional visualizations (e.g., Fig. 1G,H)^4, 21, 24, 40^. The Reich Lab COVID-19 Forecast Hub created the stimuli in this study, and this group later partnered with the CDC. The CDC used the visualizations tested in this study in their interactive COVID-19 data tracker in 2020^14^. Visualizations of only historical COVID-19 data (Fig. 1A) and those showing a mean forecast with no uncertainty (Fig. 1B) were used as controls. We tested each of the eight visualization types with cumulative and incident y-axes.

Participants, who currently live in California, were randomly assigned to one of 16 groups (*N* = 75 per group) and viewed one of the visualizations depicting current COVID-19 death data and forecasts for California, shown in Fig. 1. Participants completed the same risk judgments before and after viewing one of the visualizations. Following the primary study, participants answered questions about their demographics, COVID-19 health risk factors, and prior experiences with COVID-19. They also answered true and false questions about COVID-19^41^ and completed a brief graph literacy questionnaire^42^.

To further understand the relationship between the axis scaling (cumulative vs. incident) and the data’s trending direction (e.g., cumulative y-axes can never have downward trends vs. incident y-axes, which are omnidirectional), we conducted a follow-up study in December 2020 with participants in California and New York. At the time, California had upward trending COVID-19 death data, and New York had a flatter trend.

We used mixed-effect models to examine the proportion of variance in risk judgments accounted for by the visualization elements and participants’ characteristics. Mixed-effect models are a generalized form of linear regression, appropriate for analyzing experimental outcomes of within-subjects (in the present case, time points) and between-subjects conditions (in the present case, pre- and post-visualization exposure). Mixed-effect models are also ideal for determining the proportion of variance accounted for in risk judgments from categorical (e.g., visualization type) and continuous variables (e.g., COVID-19 knowledge). Importantly, mixed-effect models allowed us to determine the effects of our visualization manipulation on risk judgments over and beyond the meaningful impacts of (1) graph literacy, (2) COVID-19 knowledge, (3) COVID-19 health risk, (4) participants contracting COVID-19, (5) participants being tested for COVID-19, (6) age, (7) gender, and (8) education.

To test if our data met the assumptions of mixed-effect models, we used the Kolmogorov–Smirnov test to determine that the residuals were normally distributed^43^. We visually inspected the residual plots and did not observe violations from homoscedasticity or normality. Further, variance inflation factors indicated that multi-collinearity was not a problem. Our data met the assumptions for mixed-effect models. Detailed information about the analysis procedure is in the “Materials and methods”, and complete analysis in R-Markdown is in the Supplementary Information.

The main objective of this work was to determine whether COVID-19 data visualizations changed participants’ risk judgments. Therefore, we report only on the effects that involve the time points (pre- and post-visualization exposure), but full model results are available in the Supplementary Information.

**Figure 2:**
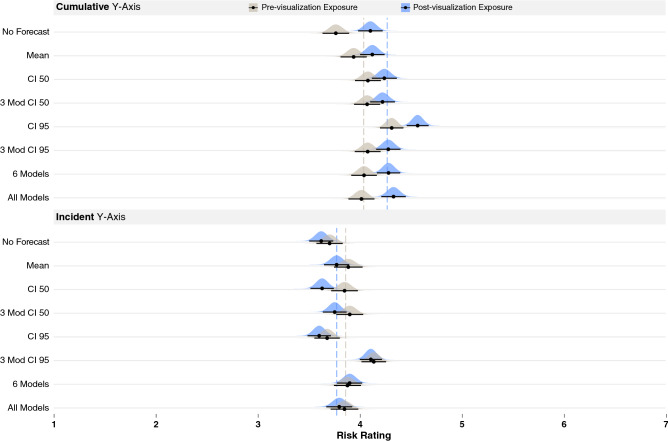
Results of Experiment 1, where pre-visualization risk judgments are colored gray and post-visualization judgments blue, for cumulative y-axis (top) and incident y-axis (bottom). Dashed lines show the mean pre- and post-visualization risk judgments for each y-axis group as a whole. Black bars show 95% confidence intervals around the mean (black dot) for each condition using the Cousineau–Morey method^44^ and the density plots were generated from this data.

### Experiment 1

The analysis revealed an interaction between time point and y-axes (*b* = − 0.33, *p* = − 0.000, *t*(1178) = − 10.48, *CIs*[− 0.39, − 0.27]). To break down this interaction, we used the same modeling procedure described above but separately analyzed the cumulative and incident y-axis groups, which suggested that visualizations with a cumulative y-axis evoked a significant increase in participants’ risk estimates (means shown in Fig. 2, top) (*b* = 0.34, *t*(582) = 5.38, *p* = 0.000, *CIs*[0.21, 0.46]). In contrast, the results from the visualizations with incident y-axes suggested they had little impact on participants’ risk estimates (means shown in the dashed lines of Fig. 2, bottom) (*b* = − 0.06, *t*(581) = − 0.99, *p* = 0.33, *CIs*[− 0.18, 0.06]). We used the same approach to break down all the interactions reported in this paper. The conditional $R^2$ for the first model was 0.57.

The second interaction revealed by the main analysis was between time points and the No Forecast vs. CI 50 visualizations (*b* = − 0.16, *t*(1178) = − 2.62, *p* = 0.009, *CIs*[− 0.29, − 0.04]). To break down this interaction, we ran separate models for the No Forecast and CI 50 visualizations. These analyses revealed a larger increase in risk estimates when participants viewed the COVID-19 line chart with no forecast (*b* = 0.33, *t*(132) = 4.79, *p* = 0.000, *CIs*[0.20, 0.47]; pre-visualization exposure *m* = 3.73, *SD* = 1.99, post-visualization exposure *m* = 3.86, *SD* = 1.83, change = 0.13) compared to the visualizations showing 50% CIs (*b* = 0.17, *t*(132) = 2.66, *p* = 0.008, *CIs*[0.05, 0.3]; pre-visualization exposure *m* = 3.97, *SD* = 1.97, post-visualization exposure *m* = 3.94, *SD* = 1.82, change = 0.03).

As a post hoc analysis, we tested if CI 50 showed less of a change in risk estimates compared to the other visualizations by running the previously described omnibus model with CI 50 as the referent. Note that the model failed to converge, and we removed time point from the random effects structure but left it in as a fixed effect to support convergence (model conditional $R^2 = 0.56$). This model revealed multiple interactions between time point and CI 50 vs. All Models, 6 Models, CI 95, 3 Models CI95, and the previously described interaction with No Forecast. The coefficients of the interactions are shown in Table 1. We ran separate models with each of the relevant visualizations to break down these interactions. The rows in Table 1 also include the main effects of time point produced by the individual models, the mean pre- and post-visualization exposure ratings, and the change in ratings. The rows are ordered according to the amount of change in risk judgments. Although all the visualizations that had interactions with CI 50 showed a meaningful increase in risk estimates, All Models had the largest change, followed by No Forecast, 6 Models, CI 95, and then 3 Models CI95. The interactions were driven by the larger changes in risk estimates produced by these visualizations compared to CI 50.

**Table 1:** Interactions between each visualization type and CI 50, along with the effects of time point when models were computed for each visualization type (ordered by size of the time point main effect). These results are collapsed across the y-axes.

	Interactions	Effect of time point	Mean risk ratings		
			Pre	Post	Change
	CI 50	*b* = 0.128, *t*(1179) = 3.274, *p* = 0.001, *CIs* [0.05, 0.20]	3.966	3.937	0.029
All models	*b* = 0.158, *t*(1179) = 3.02, *p* = 0.003, *CIs* [0.06, 0.26]	*b* = 0.314, *t*(132) = 5.69, *p* = 0.000, *CIs* [0.21, 0.42]	3.93	4.066	0.136
No forecast	*b* = 0.17, *t*(1179) = 3.18, *p* = 0.001, *CIs* [0.06, 0.27]	*b* = 0.330, *t*(132) = 4.79, *p* = 0.000, *CIs* [0.20, 0.47]	3.736	3.864	0.128
6 Models	*b* = 0.153, *t*(1179) = 2.94, *p* = 0.003, *CIs*[0.05, 0.26]	*b* = 0.237, *t*(132) = 4.50, *p* = 0.000, *CIs* [0.13, 0.34]	3.96	4.09	0.13
CI 95	*b* = 0.12, *t*(1179) = 2.31, *p* = 0.021, *CIs* [0.02, 0.22]	*b* = 0.254, *t*(131) = 5.33, *p* = 0.000, *CIs* [0.16, 0.35]	4.00	4.089	0.089
3 Model CI95	*b* = 0.11, *t*(1179) = 2.16, *p* = 0.031, *CIs* [0.01, 0.21]	*b* = 0.202, *t*(132) = 4.06, *p* = 0.000, *CIs* [0.11, 0.30]	4.108	4.195	0.087
3 Model CI 50	NA	NA	3.9	3.99	0.09
Mean	NA	NA	3.915	3.949	0.034

Finally, we noticed that CI 50 produced a negative change in risk estimates when shown with an incident y-axis (see Fig. 2, bottom third row). To determine if the CI 50 produced a reliably negative change in risk estimates compared to the other visualization types, we ran a post hoc analysis with just the incident y-axis and CI 50 as the referent. The model provided evidence for a negative impact of time point for CI 50 (*b* = − 0.22, *t*(581) = − 3.68, *p* = 0.000, *CIs* [− 0.34, − 0.11]) and interactions with 6 Models (*b* = 0.24, *t*(581) = 2.73, *p* = 0.006, *CIs* [0.07, 0.40]) and 3 Models CI95 (*b* = 0.20, *t*(581) = 2.34, *p* = 0.019, *CIs* [0.03, 0.37]). We ran follow-up analyses with the 6 Models and 3 Models CI95 as the referents, which showed little evidence for an effect of time point. These analyses suggested that participants who viewed incident y-axes with a forecast showing 50% confidence intervals significantly decreased their risk ratings compared to those who viewed forecast with 6 Models or 3 Models CI95.

**Figure 3:**
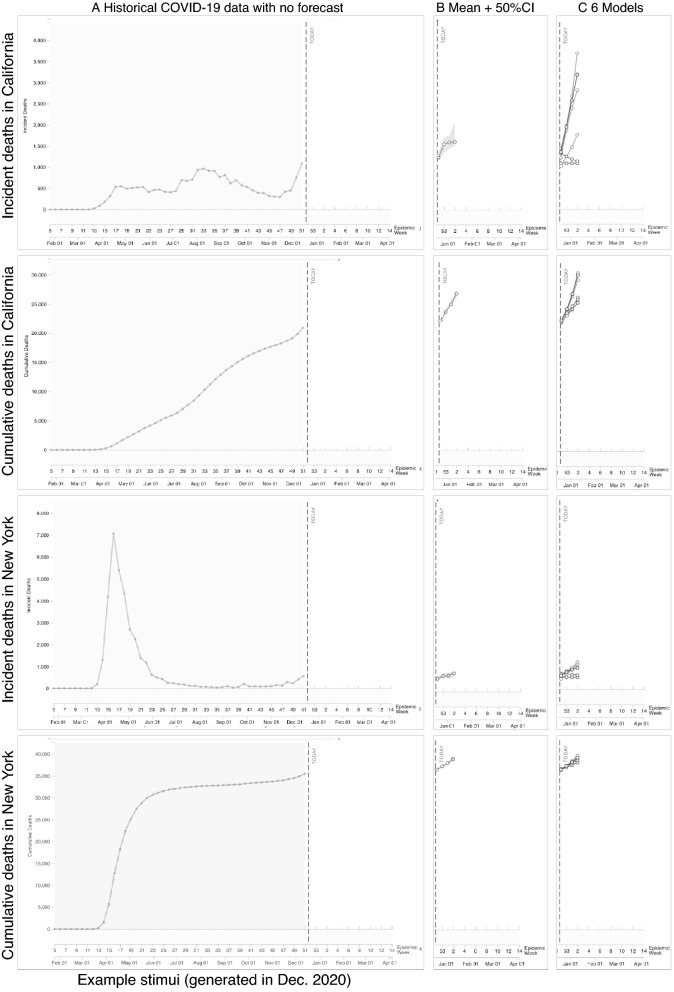
Left of figure shows line charts displaying historical COVID-19 mortality data with incident and cumulative y-axis for California and New York used in Experiment 2. Right of figure shows the visualization forecasts that were added to the data (B. CI 50 and C. 6 Models).

In sum, Experiment 1 provided evidence that cumulative y-axis scaling showing an upward trend and distributional visualizations (All Models and 6 Models) produced some of the largest increases in risk estimates. Also, CI 50 showed the smallest effects on risk judgments and was the only visualization that reduced risk estimates when paired with the incident y-axes, which showed a downward trend.

### Experiment 2

In Experiment 1, the y-axes (incident vs. cumulative) had the greatest impact on risk judgments, with the cumulative y-axis producing higher risk judgments and the incident y-axis not affecting risk judgments or sometimes producing lower risk judgments (in the case of CI 50). However, the experiment confounded the y-axis scaling with the recent trend in the data. The visualizations with an incident y-axis showed a downward trend, and those with a cumulative y-axis showed an upward trend. To understand the effect of the y-axis scaling, we ran a second experiment in December 2020 with participants in California and New York. At that time, California’s incident and cumulative y-axes showed rising trends (see Fig. 3, top two rows). If the effects in our prior experiment were due to the upward (cumulative) and downward (incident) trend of the data in the stimuli, we should see little difference between the two axes in Experiment 2, which both show upward trends. In December, no states had a downward trending incident y-axis, but New York had both incident and cumulative y-axis scaling that showed relatively flat trends (see Fig. 3, bottom two rows). We decided to conduct the same experiment with participants in New York to test if we would observe a lessened effect of time point due to the flatter upward trend of the data. To compare the y-axes across California and New York, we tested a subset of the visualizations that produced the more notable findings in Experiment 1, including No Forecast (as a control), CI 50, and 6 Models. We selected 6 Models over All Models because the 6 Models approach is more in line with visualization recommendations to reduce visual clutter. Figure 3 shows the stimuli types that we compared across the two states.

**Figure 4:**
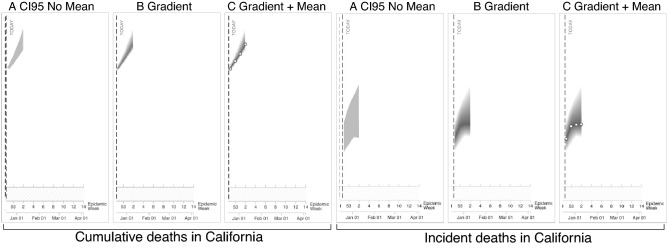
Additional uncertainty visualization techniques tested in Experiment 2 with California data.

In addition to comparing the y-axes scaling, we wanted to test three new visualization techniques that used modern visualization recommendations^21, 24^. Two of the visualizations used a Gaussian blur to convey a distribution of COVID-19 trends^24^ (referred to as *gradients*, see Fig. 4B,C)^4^. One of the gradient visualizations included a mean line (Fig. 4C), and the other did not (Fig. 4B). We also tested a CI 95 without a centerline (Fig. 4A) since recent work suggests that centerlines can bias viewers’ judgments by encouraging them to focus on the mean^21^. We tested the three new visualizations with participants only in California.

**Figure 5:**
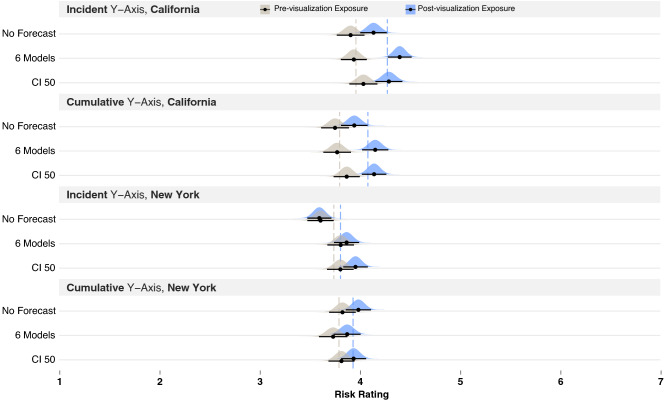
Results of the trend comparison in Experiment 2, where pre-visualization risk judgments are colored gray and post-visualization judgments blue, for cumulative y-axis (top) and incident y-axis (bottom). Dashed lines show the mean pre- and post-visualization risk judgments for each y-axis group as a whole. Black bars show 95% confidence intervals around the mean (black dot) for each condition using the Cousineau–Morey method^44^ and the density plots were generated from this data.

The first analysis used the previously described mixed-effects modeling procedure that included each of the four rows of Fig. 3 as a factor with the California incident y-axis as the referent (model conditional $R^2 = 0.51$). This analysis revealed three interactions. The first two were between time point * California incident y-axis vs. the two types of New York axes: New York incident (*b* = − 0.24, *t*(879) = − 4.69, *p* = 0.000, *CIs*[− 0.34, − 0.14]) and New York cumulative y-axis (*b* = − 0.16, *t*(879) = − 3.17, *p* = 0.002, *CIs*[− 0.26, − 0.06]; means shown in the dashed lines of Fig. 5). We ran separate models for California incident, New York incident, and New York cumulative y-axes to break down these interactions. This analysis revealed a larger effect of time point for California incident y-axis (*b* = 0.22 , *t*(207) = 3.59, *p* = 0.000, *CIs*[0.10, 0.33]) than the New York cumulative y-axis (*b* = 0.17, *t*(207) = 2.63, *p* = 0.009, *CIs*[0.04, 0.29]) and little evidence for an effect of time point for the New York incident y-axis (*b* = − 0.02, *t*(207) = − 0.26, *p* = 0.79, *CIs*[− 0.13, 0.10]). These effects can be seen in the larger difference between the dashed lines in the California incident y-axis (top of Fig. 5) compared to the smaller gaps between the dashed lines of the two types of New York axes (bottom two rows of Fig. 5). To determine if New York cumulative y-axis produced greater risk estimates than New York incident y-axis, we ran the prior omnibus model with New York cumulative y-axis as the referent. This analysis did not reveal meaningful evidence for an interaction between time point and New York cumulative vs. incident y-axis, suggesting that New York cumulative and incident y-axis evoked similar risk estimates.

The analyses in Experiment 2 suggest that the steeper upward trend of the California axes (incident and cumulative) produced larger increases in risk estimates than the flatter trend of the New York data. In sum, the results of Experiment 1—where the cumulative y-axes affected risk perceptions whereas the incidence y-axes did not—were likely driven by the trends in the y-axis. To test if our assumptions about the relative slopes of the data in the stimuli were correct, we measured the angles of the lines in the stimuli two weeks before the forecast. We confirmed that the California incident y-axis had the steepest line angle (67$^{\circ }$), followed by California cumulative (44$^{\circ }$), New York incident (34$^{\circ }$), and New York cumulative (21$^{\circ }$). In line with Experiment 1, this analysis suggested that the 6 Models visualization produced the largest increases in risk estimates, which were significantly larger than the No Forecast visualization.

**Figure 6:**
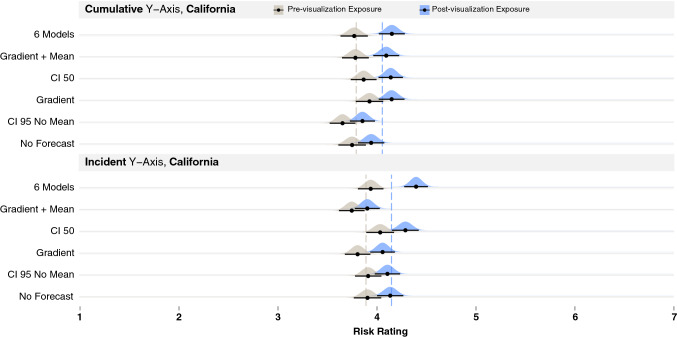
Results of the visualization comparison in Experiment 2 (ordered by size of the time point main effect), where pre-visualization risk judgments are colored gray and post-visualization judgments blue, for cumulative y-axis (top) and incident y-axis (bottom). Dashed lines show the mean pre- and post-visualization risk judgments for each y-axis group as a whole. Black bars show 95% confidence intervals around the mean (black dot) for each condition using the Cousineau–Morey method^44^ and the density plots were generated from this data.

The goal of the second analysis was to examine the impact of the three new visualization techniques (see Fig. 4) that participants in California viewed. We specified 6 Models as the referent because they showed the largest increase in risk ratings in the last analysis. This analysis revealed interactions between time point and 6 Models vs. all the other visualizations (see Table 2 for interaction coefficients) (model conditional $R^2 = 0.50$). To break down these interactions, we ran separate models for each visualization type. The models other than the 6 Models and No Forecast failed to converge, and we removed time point as a random slope from the model to support convergence. Table 2 shows the fixed effect of time point for each visualization type along with mean pre- and post-visualization exposure risk ratings. This analysis revealed that although each of the visualizations showed a meaningful increase in risk estimates, the 6 Models visualization produced significantly larger increases in risk estimates (mean change of 0.43) than each of the other visualization techniques (mean change of 0.23 on average). Figure 6 shows the larger increase in risk estimates for 6 Models, and the techniques are ordered by their average change.

Taken together, the results of Experiment 1 and 2 provide evidence that cumulative y-axes that more likely show an upward trend and COVID-19 visualizations that show six or more models relatively consistently evoke the largest increases in risk estimates compared to the other COVID-19 visualization techniques tested in this work.

**Table 2:** Interactions between each visualization type and 6 Models, along with the effects of time point when models were computed for each visualization type (ordered by the size of the time-point main effect).

	Interactions	Effect of time point	Mean risk ratings		
			Pre	Post	Change
	6 Models	*b* = 0.428, *t*(879) = 9.41, *p* = 0.000, *CIs* [0.34,0.52]	3.857	4.27	0.413
Gradient with mean	*b* = − 0.199, *t*(879) = − 3.34, *p* = 0.001, *CIs* [− 0.32, − 0.08]	*b* = 0.306, *t*(133) = 5.61, *p* = 0.000, *CIs* [0.20, 0.41]	3.768	4.00	0.232
CI 50	*b* = − 0.175, *t*(879) = − 2.92, *p* = 0.004, *CIs* [− 0.29, − 0.06]	*b* = 0.267, *t*(133) = 4.86, *p* = 0.000, *CIs* [0.16, 0.37]	3.95	4.21	0.26
Gradient	*b* = − 0.180, *t*(879)= − 3.02, *p* = 0.003,*CIs* [− 0.30, − 0.06]	*b* = 0.226, *t*(133) = 4.16, *p* = 0.000, *CIs* [0.12, 0.33]	3.87	4.11	0.24
CI 95 No mean	*b* = − 0.222, *t*(879) = − 3.71, *p* = 0.000, *CIs* [− 0.34, − 0.11]	*b* = 0.201, *t*(133) = 3.65, *p* = 0.000, *CIs* [0.09, 0.31]	3.786	3.98	0.194
No forecast	*b* = − 0.216, *t*(879) = − 3.60, *p* = 0.000, *CIs* [− 0.33, − 0.10]	*b* = 0.190, *t*(133) = 3.23, *p* = 0.001, *CIs* [0.08, 0.31]	3.83	4.04	0.21

## Discussion

Scholars have suggested that the COVID-19 pandemic created a “breakthrough moment” in data visualization^45^, where millions around the world turned to visualizations to understand their health risks^2^. The results from our studies suggest that viewing visualizations, like those used by the CDC in 2020 to convey COVID-19 data, fundamentally changes people’s pandemic risk perceptions. Importantly, this work provides evidence that visualizations can influence risk perceptions over and beyond the impacts of gender, age, education, prior experiences with COVID-19, knowledge about COVID-19, and health risks for COVID-19. Further, we found that visualizing the same data in different ways had differential effects on the changes we observed in risk perceptions. Our work suggests that using a cumulative y-axis will most reliably lead to people perceiving greater pandemic risk because cumulative y-axis can show only an upward or flat trend. In contrast, incident y-axis can be highly variable and even reduce viewers’ perceived pandemic risk, if the charts exhibit a downward trend. An incident y-axis is the default on the CDC’s website^14^ at the time of publication. We found that significant increases and decreases in perceived risk can be achieved simply by changing the y-axis without any modifications to the underlying data or forecast predictions. This finding emphasizes the importance of informing risk communicators of the implications of visualization choices. Without evidence-based guidance, risk communicators could create visualizations that inadvertently contribute to a public misinterpretation of pandemic risks.

Additionally, we found that uncertainty pandemic forecasts that showed distributional information in the form of multiple forecast models (i.e., ensembles of six or more models) tended to produce the largest increases in risk estimates. The impact of the 6 Models and All models visualizations is consistent with research demonstrating the efficacy of ensembles^13, 37, 39, 46^ and distributional uncertainty visualizations (e.g.,^21, 24, 35, 36, 38^). The results also suggested that 50% confidence intervals were the least likely to change risk perceptions and sometimes decreased risk perceptions. The majority of active COVID-19 visualizations use confidence intervals (70%^1^), including the default visualization on the CDC’s website^14^ at the time of publication. Extensive use of confidence intervals should be reevaluated because they tend to produce variable changes in risk perceptions, which may not be desirable when conveying health risks to the public. Together, these findings provide evidence that visualizations that depict forecasts with uncertainty can meaningfully change people’s beliefs about health risks, as well as illustrate the utility of data visualizations in risk communication during pandemic events.

This work also has several limitations. For one, there was no clear correct answer for the risk estimates. We opted to use Likert scales to evaluate relative risk estimates rather than asking people to estimate probability because reasoning with probability is challenging for most of the public^47^. However, we cannot report if visualization techniques make the participants’ risk judgments more accurate without a correct answer. Also, we cannot assert that the changes we observed in risk estimates lead to changes in behavior. Instead, our work focuses on the relative change in risk judgments as a critical component in determining the impact of visualizations on people’s understanding of risk. Additionally, although we tested many data visualization types (34 visualizations total), this work did not comprehensively study every modern uncertainty visualization approach (for reviews, see^31, 48^). Further, while we included eight individual differences measures, which we selected based on prior COVID-19 research, we did not exhaustively test every individual differences measure that may have influenced participants’ risk judgments. Our conclusions, therefore, are limited to the visualizations and individual differences measures examined in our studies. It is also important to note that we included one-sentence descriptions of the visualizations on each trial, which could have influenced the observed effects and are available in the Supplementary Information (page 10). Examples include “The right of the figure shows a COVID-19 projection with a 95% confidence interval” and “The right of the figure shows 6 COVID-19 projections.” Additional research is needed to determine the optimal descriptions for uncertainty visualizations.

In closing, this work provides evidence that visualizations of pandemic data can fundamentally change COVID-19 risk perceptions. We recommend that risk outreach organizations consider the impacts of y-axes scales that produce variable risk perceptions based on the recent pandemic trends. Further, risk communicators should select a method for visualizing time series forecast data tailored to their desired public response. Risk communicators must decide how these influential instruments should be used and calibrated.

## Materials and methods

The experiments were conducted online with populations of participants from Prolific^49^, which was census matched to reflect population characteristic distributions of the United States. The experiments took roughly 15 mins to complete, and the participants were paid $3. The prescreening criteria dictated that participants were over 18 years old, lived in either California or New York, and were not allowed to participate in both experiments.

### Approval for human experiments

The Institutional Review Board of the University of California Merced reviewed the protocol of these experiments and approved them for human subjects research. Both experiments were performed in accordance with the University of California Merced guidelines. Informed consent was obtained from all participants prior to completing the study.

### Procedure

Before beginning the studies, participants consented via an IRB consent protocol and were instructed not to participate in the experiment with a screen smaller than 9.4 $\times$ 6.6 inches. Participants then read the following instructions:

*“In the first section, you will be asked to make judgments about COVID-19 risk. Some questions will ask you to make judgments about yourself, and some questions will ask you to make judgments about other people. For example, one question might be: How impactful is COVID-19 to the daily lives of the following people? Click on the option below that best describes your beliefs for each person.”* Below the instructions, a Likert scale was included that ranged from 1 (definitely not impactful) to 7 (definitely impactful).

Following the instructions, participants answered four questions (referred to as *question types*) on a 1–7 Likert scale that included number and word anchors based on recommendations from^50^. Participants answered the four questions below about (1) themselves, (2) an average 22-year-old in their state who is generally in good health, and (3) an average 78-year-old in their state who is generally in good health (referred to as *target type*).

1. How often do you think each of the following people will come in contact with someone currently infected with COVID-19 in the next 2 weeks? Scale: Never (1), Very seldom (2), Rather infrequently (3), Some of the time (4), Fairly often (5), Very frequently (6), Constantly (7)
2. What is the risk that the following people will contract COVID-19 within the next 2 weeks? Scale: No risk (1) A small degree of risk (2), A limited amount of risk (3), Some risk (4), A good bit of risk (5), A great deal of risk (6), Complete risk (7).
3. Imagine the following people contracted COVID-19, how many of their symptoms do you think will be severe? Scale: No severe symptoms (1), A small degree of severe symptoms, (2), A limited amount of severe symptoms (3), Some severe symptoms (4), A good bit of severe symptoms (5), A great deal of severe symptoms (6), All severe symptoms (7).
4. Imagine the following people contracted COVID-19. What is their risk of experiencing adverse effects that would require hospitalization? Scale: No risk (1), A small degree of risk (2), A limited amount of risk (3), Some risk (4), A good bit of risk (5), A great deal of risk (6), Complete risk (7).

The participants were then given brief instructions about the components of the visualizations that corresponded to the axis they were randomly assigned to view. These instructions were comprised of an example figure that was not one of the stimuli used in the study. The example figure highlighted the historical COVID-19 data and included the annotation “cumulative number of deaths in your state since the pandemic started” or “number of deaths per week in your state since the pandemic started”. On the next screen, a figure highlighted the gap between the historical data and predictions reading, “delay between data reporting and prediction.” On the last instruction screen, the location where the COVID-19 forecast would be placed was highlighted, and an annotation was included reading, “You may see a 2-week COVID-19 prediction here.” No additional instructions were given based on the uncertainty visualizations to ensure that differences between instructions did not drive the results in the study.

The participants then viewed the visualization that they were randomly assigned to for 1.5 min before being allowed to progress to the next screen. After viewing the visualization, the participants repeated the risk judgments. They then completed a series of follow-up questions, which included a four-item graph literacy questionnaire^42^, 13 true and false COVID-19 knowledge questions^41^, 20 COVID-19 health risk factors identified by the CDC^51^, if they had been tested for COVID-19, if they were diagnosed with COVID-19, if they thought they had COVID-19 but did not get diagnosed, and their age, gender, and education level.

### Experiment 1 sample and stimuli

Participants were 1200 California residents (45.37% Women,  *M* age = 32.74 years, *SD* = 11.90 years). Experiment 1 used a mixed design: between − 2 (cumulative vs. incident y-axis) $\times$ 8 (visualization techniques, see Fig. 1) and within-subjects 2 (pre- and post-visualization exposure) $\times$ 3 (judgment target) $\times$ 4 (question type). The 75 participants were randomly assigned to each of the 16 groups. Using prior research on decision making with visualizations as pilot data, we calculated an anticipated effect size (pseudo *r*^2^ = 0.02) and used the program G*Power^52^ to determine we would need 75 participants per visualization group. One participant in the group that viewed the CI 95 visualization experienced browser issues and was removed from the analyses, resulting in  *N* = 1199. There were no meaningful differences for the individual differences measures between the groups.

The full stimuli set, instructions, procedure, and question text are available in the Supplemental Materials. Figure 1 (H) shows 35 forecast models that different research groups produced with varied input data and assumptions concerning COVID-19 mitigation measures^53^. These models were used to create the mean forecast (Fig. 1B) and the 50% (Fig. 1C) and 95% confidence interval visualizations (Fig. 1E). The mean forecast was a secondary control that showed a forecast but no uncertainty. D and F in Fig. 1 each show three models selected to match the range of the 50% or 95% confidence intervals. Our goal in using the three-model approach was to understand if the effects of the confidence intervals were driven by the differences in visual salience of the filled-in intervals or by the physical span of the intervals. Figure 1G shows six models with minimal model assumptions that the modeling specialist in our group selected. The experiment showed participants the stimuli with a label indicating the state and y-axes, along with a one-sentence caption describing the forecast visualization. The descriptions ranged from 6 to 14 words and are available in the supplementary materials along with the instructions. The descriptions were intentionally minimal to simulate how forecasters commonly present model results. For example, our prior work found that of the 20 most viewed TV forecasts of hurricane Irma in 2017, the average length of the forecast was 1:52 mins and no forecasters described how they created the visualizations^19^.

### Experiment 2 sample and stimuli

Participants were 675 California residents (50.11% Women,  *M* age = 30.93 years, *SD* = 11.83 years) and 675 New York residents (45.75% Women,  *M* age = 32.26 years, *SD* = 11.69 years). A mixed design was used for the first comparison in Experiment 2: between − 2 (cumulative vs. incident y-axis) $\times$ 2 (Californian vs. New York) $\times$ 3 (visualization techniques, see Fig. 3) and within-subjects 2 (pre- and post-visualization exposure) $\times$ 3 (judgment target) $\times$ 4 (question type). This design created 12 groups with 75 participants randomly assigned from California to each group (*N* = 900). Additional between-subjects groups were added to Experiment 2 to examine the effect of three new visualizations (see Fig. 4). There were no meaningful differences for the individual differences measures between the groups.

### Statistical analysis

We computed the results presented in this paper using the programming language R^54^ and the following R packages for the analysis: *tidyverse* v. 1.3.1^55^ (data processing), *ggdist* v. 3.0.1^56^ (visualization), *lme4* v. 1.1-27.1^57^ (mixed-effects modeling), and *MuMIn* v. 1.43.17^58^ (effects size estimation). As described in the Results section, we selected linear mixed models to examine our hypotheses, and the lme4 package fits linear mixed models using maximum likelihood. P-values were estimated using the Wald method. We based the models on the empirical questions in this work and prior research indicating the impact of the included individual differences measures on uncertainty judgments. We specified risk estimates as the outcome variable, which are ordinal but were treated as continuous because they contained more than five response categories^59^.

As shown in the lmer model syntax below, for the primary Model 1 in Experiment 1, we included the following fixed effects: *Time Point* (pre- vs. post-visualization exposure, with pre as the referent), *Y-axes* (cumulative vs. incident California death data, with cumulative as the referent), *Visualization* (eight visualization techniques, with the No Forecast visualization as the referent) and the two-way interactions between each of these factors as predictors. We also included the variants of the risk judgments and individual differences measures as covariates, which were fixed effects: *Question Type* (four risk questions), *Question Target* (self, younger and older adult). Each of the following individual differences were either dummy coded (for categorical variables) or mean centered (for continuous variables): *Graph Literacy* (centered), *COVID-19 Knowledge* (centered), *COVID-19 Health Risk* (centered), previously *Contracted COVID-19* (dummy coded), previously *Tested for COVID-19* (dummy coded), *Age* (centered), *gender* (dummy coded), and *Education* (centered). The results of the visualization manipulations that we report in the Results section are over and beyond the effects of these covariates (see Fig. 2 for results). We also included random intercepts for each participant and random slopes for *Time Point*, *Question Type*, and *Judgment Target*.

Experiment 1, lmer model syntax:
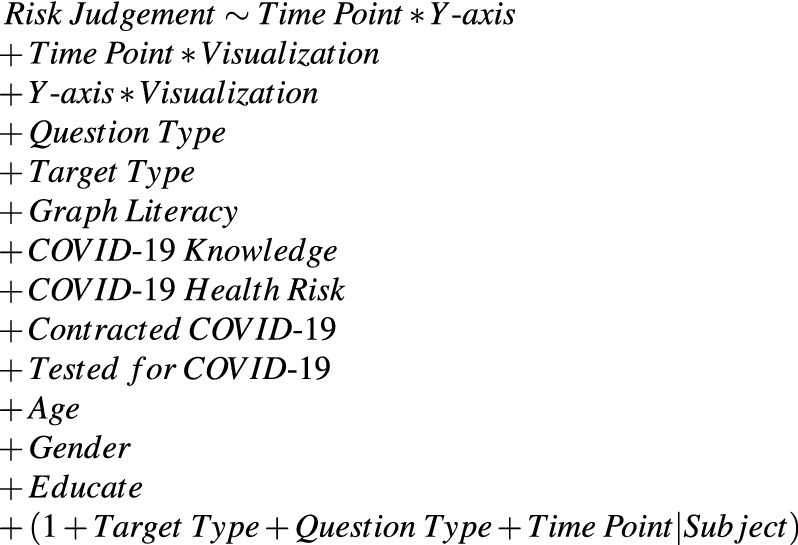
We used the same modeling procedure for the first analysis in Experiment 2 as in Experiment 1. The only differences were that we replaced the y-axis variable with a variable indicating the state and y-axis combination (California incident y-axis as the referent compared to California cumulative y-axis, New York incident y-axis, and New York cumulative y-axis), and we analyzed three visualization types (No forecast as the referent compared to 6 Models and CI 50) (see Fig. 5 for results). For the second analysis in Experiment 2, we focused on the data from participants in California and used the same model as in Experiment 1 but with six visualization types (6 Models as the referent compared to No Forecast, CI 50, Gradient, Gradient With Mean, and CI 95 No Mean) (see Fig. 6 for results).

## Supplementary Information

Supplementary Information.

## Data Availability

The data and complete analysis R markdown script are available on the Open Science Framework (https://osf.io/hfvqc/).
