# Publisher Correction: Impact of COVID-19 forecast visualizations on pandemic risk perceptions

**DOI:** 10.1038/s41598-022-07502-y

**Published:** 2022-03-01

**Authors:** Lace Padilla, Helia Hosseinpour, Racquel Fygenson, Jennifer Howell, Rumi Chunara, Enrico Bertini

**Affiliations:** 1 Cognitive and Information Sciences Department, University of California Merced, Merced, 95340 USA; 2 Computer Science and Engineering Department, New York University, New York, 748766 USA; 3 Psychological Sciences Department, University of California Merced, Merced, 95340 USA; 4 Computer Science and Engineering Department, Northeastern University, Boston, 02115 USA

Correction to: Scientific Reports 10.1038/s41598-022-05353-1, published online 07 February 2022

The original version of this Article contained an error in Figure 6 where “No Forecast” was incorrectly given as “No Rorecast”.

The original Figure 6 accompanying legend appears below.

**Figure 6:**
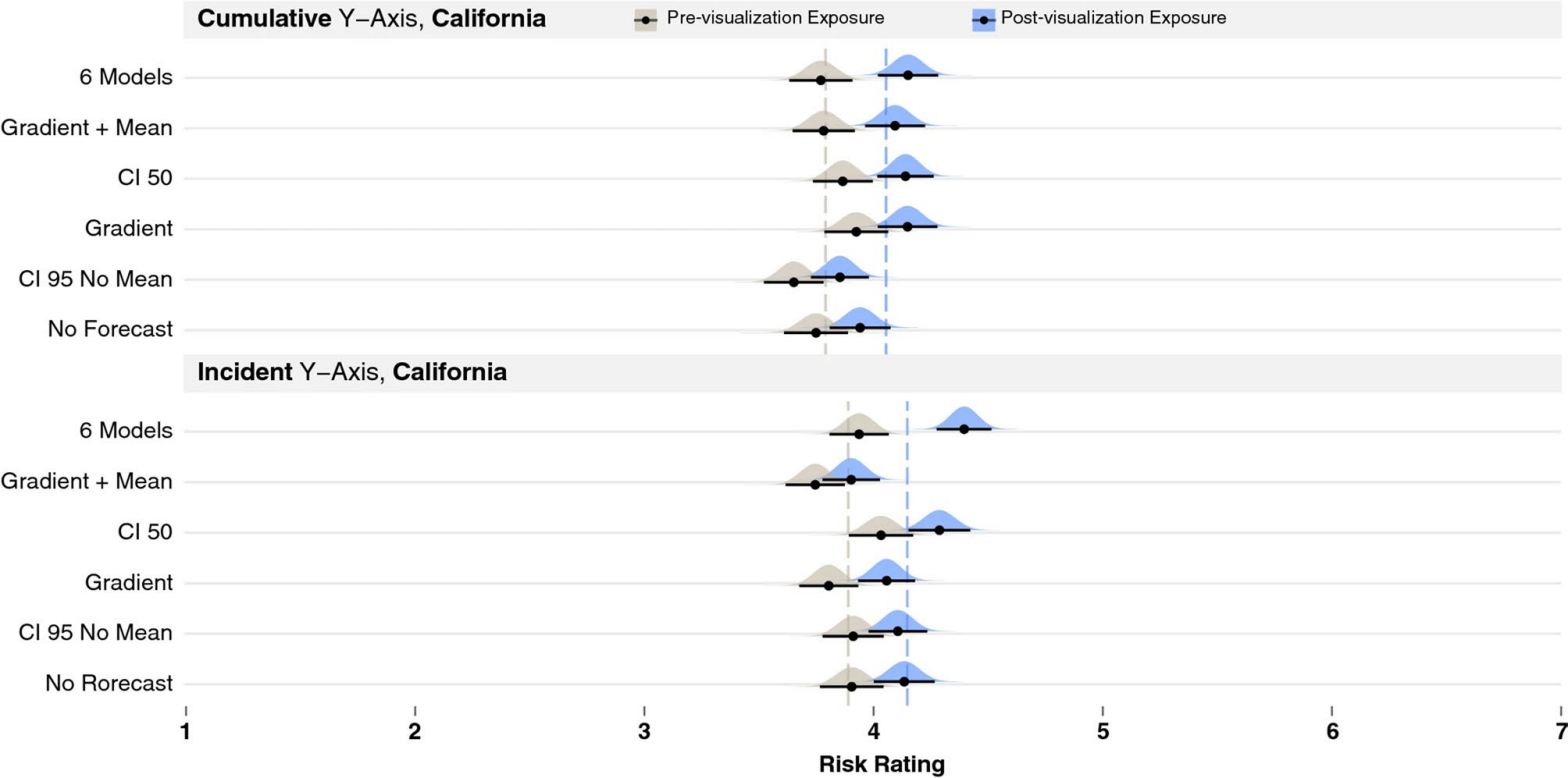
Results of the visualization comparison in Experiment 2 (ordered by size of the time point main effect), where pre-visualization risk judgments are colored gray and post-visualization judgments blue, for cumulative y-axis (top) and incident y-axis (bottom). Dashed lines show the mean pre- and post-visualization risk judgments for each y-axis group as a whole. Black bars show 95% confidence intervals around the mean (black dot) for each condition using the Cousineau–Morey method^44^ and the density plots were generated from this data.

The original Article has been corrected.

