# Supplementary material for: Impact of COVID-19 forecast visualizations on pandemic risk perceptions
Source: Sci Rep. 2022 Feb 7;12:2014. doi: 10.1038/s41598-022-05353-1 (PMC8821632; doi:10.1038/s41598-022-05353-1)
Supplement: Supplementary file 1 — Supplementary Information. [file 41598_2022_5353_MOESM1_ESM.pdf]

Supplementary materials: Impact of COVID-19 Forecast Visualizations on Pandemic Risk Perceptions

Contents

1 Summary of participant characteristics for each experiment and condition 2
1.1 In each experiment 2
1.1.1 Gender, age, higher education, and graph literacy 2
1.1.2 COVID-19 knoweldge, COVID-19 health risk factors, and COVID-19 experience 2
1.2 In each between-subjects condition 3
1.2.1 Gender, age, higher education, and graph literacy 3
1.2.2 COVID-19 knoweldge, COVID-19 health risk factors, and COVID-19 experience 4

2 Procedure 5
2.1 Primary task 5
2.2 Screenshots of instructions and primary task 6
2.3 COVID-19 knowledge questions from Azlan et al. (2020) 7
2.4 Graph literacy questions from Okan et al. (2019) 8
2.5 COVID-19 health risk factors (CDC) 9

3 Stimuli 10
3.1 Exp 1 10
3.2 Exp 2 12

4 Analysis 13
4.1 Exp 1 13
4.1.1 Model 1 Omnibus 13
4.1.2 Plot of primary task results 15
4.1.3 Interaction analysis: Time point * Y-axis 15
4.1.4 Interaction analysis: Time point * No Forecast vs. CI 50 16
4.1.5 Post hoc test: Full model CI 50 as the referent 17
4.1.6 Post hoc test: Negative effect of time point for CI 50 incident data 20
4.2 Exp 2 22
4.2.1 Model 1 (CA vs. NY) 22
4.2.2 Plot of primary task results 23
4.2.3 Interaction analysis: Time point * CA cumulative, NY incident, NY cumulative 24
4.2.4 Interaction analysis: Time Point * No Forecast vs. 6 Models 26
4.2.5 Model 2 (CA additional visualizations) 27
4.2.6 Plot of primary task results 28
4.2.7 Interaction analysis: Time Point * 6 Models vs. all other visualizations 29

5 Data and code availability 31

1 Summary of participant characteristics for each experiment and condition

1.1 In each experiment

1.1.1 Gender, age, higher education, and graph literacy

				Age				Graph literacy	
**Exp**	**State**	***N***	**women (%)**	**mean**	***SD***	**min-max**	**higher-ed (%)**	**mean**	***SD***
Exp1	CA	1199	45.37	32.74	11.90	18-77	66.06	2.16	1.20
Exp2	CA	900	50.11	30.93	11.82	18-81	55.78	2.35	1.11
Exp2	NY	450	45.78	32.26	11.69	18-78	66.44	2.18	1.12

Note: Exp = The experiment each participant was in. State = Resident state of the online participants. N = number of participants per condition. % women = the proportion of women in each experiment. Age mean = the average age of participants and SD is the mean age standard deviation. Age range = the min and max ages of participants. Higher ed = the proportion of participants who completed an undergraduate, master's, Ph.D., or equivalent. Graph literacy mean = the average graph literacy score base on Okan et al (2019) and SD is the standard deviation of the mean graph literacy.

1.1.2 COVID-19 knoweldge, COVID-19 health risk factors, and COVID-19 experience

		COVID-19							
		Knowledge scale(0-13)		Health risk scale(0-20)		Direct Experience			
**Exp**	**State**	**mean**	***SD***	**mean**	***SD***	**Diagnosed (%)**	**Tested (%)**	**Undiagnosed but had (%)**	**Awaiting Results (%)**
Exp1	CA	10.02	1.91	0.78	1.31	6.92	35.45	14.35	7.26
Exp2	CA	10.10	1.66	0.58	0.90	1.78	37.33	11.11	3.11
Exp2	NY	10.23	1.61	0.62	0.94	3.33	48.89	12.89	1.56

Note: COVID knowledge mean and SD = Azlan et al. (2020; possible scores range from 0-13). COVID-19 Risk Factors mean and SD = list of 20 COVID-19 health risk factors from the CDC. The value of 1 was assigned to each item (possible scores range from 0 - 20). Diagnosed = percent of each group who had been diagnosed with COVID-19 at any point in the past. Tested = percent of each group who has been tested for COVID-19. Undiagnosed but had = percent of each group who believed they had COVID-19 in the past but were not diagnosed. Awaiting Results = percent of each group who were awaiting their test results.

1.2 In each between-subjects condition

1.2.1 Gender, age, higher education, and graph literacy

Exp	State	Stimuli (Group)	*N*	women (%)	Age			higher-ed (%)	Graph literacy	
					mean	min-max	*SD*		mean	*SD*
Exp1	CA	3 Mod CI50	150	47.33	34.50	18-72	12.79	75.33	1.99	1.30
Exp1	CA	3 Mod CI95	150	40.67	33.86	18-68	11.94	68.00	2.19	1.19
Exp1	CA	6 Models	150	44.67	32.88	18-68	11.77	65.33	2.13	1.19
Exp1	CA	All Models	150	52.00	32.85	18-71	11.99	60.67	2.26	1.17
Exp1	CA	CI 50	150	46.00	31.33	18-73	10.33	70.00	2.15	1.25
Exp1	CA	CI 95	149	41.61	35.04	18-72	13.55	67.79	1.95	1.16
Exp1	CA	Mean	150	45.33	31.29	18-73	11.25	62.00	2.29	1.18
Exp1	CA	No Forecast	150	45.33	30.14	18-77	10.68	59.33	2.29	1.13
Exp2	CA	6 Models	150	50.00	30.77	18-73	11.24	56.67	2.29	1.03
Exp2	CA	CI 50	150	47.33	30.69	18-81	11.27	59.33	2.26	0.99
Exp2	CA	CI95 No Mean	150	50.00	30.82	18-81	12.70	53.33	2.40	1.15
Exp2	CA	Gradient	150	54.67	30.29	18-73	11.26	58.67	2.46	1.19
Exp2	CA	Gradient+Mean	150	49.33	32.89	18-77	13.67	51.33	2.28	1.15
Exp2	CA	No Forecast	150	49.33	30.13	18-72	10.47	55.33	2.42	1.15
Exp2	NY	6 Models	150	50.00	32.78	18-72	11.93	65.33	2.28	1.08
Exp2	NY	CI 50	150	48.00	31.92	18-70	11.47	66.00	1.99	1.07
Exp2	NY	No Forecast	150	39.33	32.07	18-78	11.72	68.00	2.25	1.19

Note: Exp = The experiment each participant was in. State = Resident state of the online participants. *N* = number of participants per condition. % women = the proportion of women in each experiment. Age mean = the average age of participants and *SD* is the mean age standard deviation. Age range = the min and max ages of participants. Higher ed = the proportion of participants who completed an undergraduate, master's, Ph.D., or equivalent. Graph literacy mean = the average graph literacy score base on Okan et al (2019) and *SD* is the standard deviation of the mean graph literacy.

1.2.2 COVID-19 knoweldge, COVID-19 health risk factors, and COVID-19 experience

			COVID-19							
			Knowledge		Health risk		% Direct Experience			
Exp	State	Stimuli (Group)	mean	*SD*	mean	*SD*	Diagnosed	Tested	Undiag-nosed but had	Awaiting Results
Exp1	CA	3 Mod CI50	10.03	1.83	0.73	0.96	9.33	35.33	14.67	8.00
Exp1	CA	3 Mod CI95	9.87	2.04	1.06	2.28	10.00	38.00	15.33	12.67
Exp1	CA	6 Models	10.11	1.99	0.75	1.21	6.00	38.00	16.00	5.33
Exp1	CA	All Models	10.34	1.50	0.77	1.05	2.67	26.67	15.33	2.67
Exp1	CA	CI 50	9.85	1.97	0.80	1.14	8.67	40.67	12.67	9.33
Exp1	CA	CI 95	9.73	2.20	0.91	1.28	14.77	36.24	18.79	13.42
Exp1	CA	Mean	10.14	1.66	0.69	1.08	2.67	36.67	9.33	3.33
Exp1	CA	No Forecast	10.09	1.97	0.57	0.96	1.33	32.00	12.67	3.33
Exp2	CA	6 Models	9.93	1.59	0.54	0.84	3.33	38.67	9.33	2.00
Exp2	CA	CI 50	10.23	1.59	0.53	0.91	0.67	40.00	12.67	3.33
Exp2	CA	CI95 No Mean	10.09	1.62	0.69	0.98	0.67	38.00	16.00	4.00
Exp2	CA	Gradient	10.24	1.32	0.61	0.99	0.67	32.00	11.33	2.67
Exp2	CA	Gradient+Mean	10.11	1.99	0.59	0.87	3.33	29.33	6.00	2.67
Exp2	CA	No Forecast	10.02	1.75	0.53	0.81	2.00	46.00	11.33	4.00
Exp2	NY	6 Models	10.30	1.57	0.68	1.07	4.67	48.67	12.00	1.33
Exp2	NY	CI 50	10.29	1.61	0.61	0.86	2.00	53.33	14.00	0.67
Exp2	NY	No Forecast	10.09	1.63	0.56	0.87	3.33	44.67	12.67	2.67

Note: COVID knowledge mean and *SD* = Azlan et al. (2020; possible scores range from 0-13). COVID-19 health risk factors mean and *SD* = list of 20 COVID-19 health risk factors from the CDC. The value of 1 was assigned to each item (possible scores range from 0 - 20). Diagnosed = percent of each group who had been diagnosed with COVID-19 at any point in the past. Tested = percent of each group who has been tested for COVID-19. Undiagnosed but had = percent of each group who believed they had COVID-19 in the past but were not diagnosed. Awaiting Results = percent of each group who were awaiting their test results.

2 Procedure

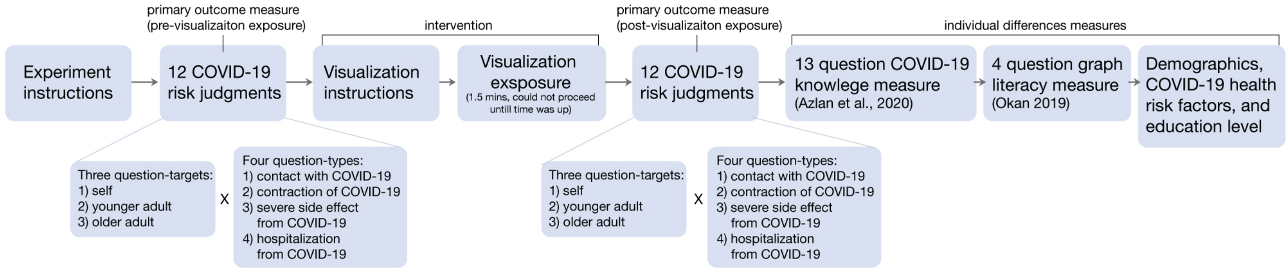

2.1 Primary task

In experiments 1 and 2, participants were asked to answer the four question about 1) themselves, 2) an average 22-year-old in their state, who is generally in good health, and 3) an average 78-year-old in their state, who is generally in good health. Participants answered all of these questions before and after view a COVID-19 data visualization.

Risk_Question	Anchor1	Anchor2	Anchor3	Anchor4	Anchor5	Anchor6	Anchor7
1) How often do you think each of the following people will come in contact with someone currently infected with COVID-19 in the next two weeks?	Never (1)	Very seldom (2)	Rather infrequently (3)	Some of the time (4)	Fairly often (5)	Very frequently (6)	Constantly (7)
2) What is the risk that the following people will contract COVID-19 within the next two weeks?	No risk (1)	A small degree of risk (2)	A limited amount of risk (3)	Some risk (4)	A good bit of risk (5)	A great deal of risk (6)	Complete risk (7)
3) Imagine the following people contracted COVID-19, how many of their symptoms do you think will be severe?	No severe symptoms (1)	A small degree of severe symptoms (2)	A limited amount of severe symptoms (3)	Some severe symptoms (4)	A good bit of severe symptoms (5)	A great deal of severe symptoms (6)	All severe symptoms (7)
4) Imagine the following people contracted COVID-19. What is their risk of experiencing adverse effects that would require hospitalization?	No risk (1)	A small degree of risk (2)	A limited amount of risk (3)	Some risk (4)	A good bit of risk (5)	A great deal of risk (6)	Complete risk (7)

2.2 Screenshots of instructions and primary task

Screen 1

Section 1 Instructions:
In the first section, you will be asked to make judgments about COVID-19 risk. Some questions will ask you to make judgments about yourself, and some questions will ask you to make judgments about other people.

For example, one question might be:
How impactful is COVID-19 to the daily lives of the following people?
Click on the option below that best describes your beliefs for each person.

	**definitely not** impactful (1)	**probably not** impactful (2)	**maybe not** impactful (3)	**neutrical** impactful (4)	**maybe** impactful (5)	**probably** impactful (6)	**definitely** impactful (7)
**You**	◯	◯	◯	◯	◯	◯	◯
**An average person in California**	◯	◯	◯	◯	◯	◯	◯

Once you have made your judgment, click the next button.

Screen 2

1) How often do you think each of the following people will **come in contact** with someone currently infected with COVID-19 in the next two weeks?

	Never (1)	Very seldom (2)	Rather infrequently (3)	Some of the time (4)	Fairly often (5)	Very frequently (6)	Constantly (7)
**You**	◯	◯	◯	◯	◯	◯	◯
An average **22**-year-old in **California**, who is generally in good health	◯	◯	◯	◯	◯	◯	◯
An average **78**-year-old in **California**, who is generally in good health	◯	◯	◯	◯	◯	◯	◯

Screen 3

2) What is the risk that the following people will **contract COVID-19** within the next two weeks?

	No risk (1)	A small degree of risk (2)	A limited amount of risk (3)	Some risk (4)	A good bit of risk (5)	A great deal of risk (6)	Complete risk (7)
**You**	◯	◯	◯	◯	◯	◯	◯
An average **22**-year-old in **California**, who is generally in good health	◯	◯	◯	◯	◯	◯	◯
An average **78**-year-old in **California**, who is generally in good health	◯	◯	◯	◯	◯	◯	◯

Screen 4

3) Imagine the following people contracted COVID-19, **how many of their symptoms do you think will be severe**?

	No severe symptoms (1)	A small degree of severe symptoms (2)	A limited amount of severe symptoms (3)	Some severe symptoms (4)	A good bit of severe symptoms (5)	A great deal of severe symptoms (6)	All severe symptoms (7)
**You**	◯	◯	◯	◯	◯	◯	◯
An average **22**-year-old in **California**, who is generally in good health	◯	◯	◯	◯	◯	◯	◯
An average **78**-year-old in **California**, who is generally in good health	◯	◯	◯	◯	◯	◯	◯

Screen 5

4) Imagine the following people contracted COVID-19. What is their risk of experiencing adverse effects that would **require hospitalization**?

	No risk (1)	A small degree of risk (2)	A limited amount of risk (3)	Some risk (4)	A good bit of risk (5)	A great deal of risk (6)	Complete risk (7)
**You**	◯	◯	◯	◯	◯	◯	◯
An average **22**-year-old in **California**, who is generally in good health	◯	◯	◯	◯	◯	◯	◯
An average **78**-year-old in **California**, who is generally in good health	◯	◯	◯	◯	◯	◯	◯

Screen 6

Section 2 Instructions:
In the second section, you will be asked to view a graph for 1.5 mins, which will show COVID-19 information, like the one below.

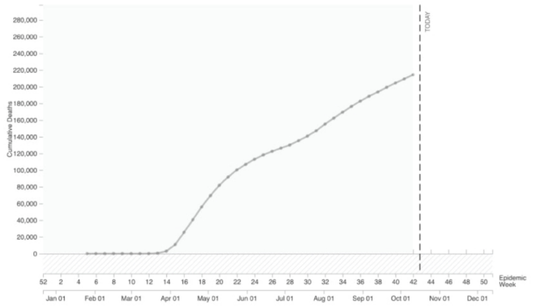

Screen 7

Section 2 Instructions:
Here is how to read these graphs:

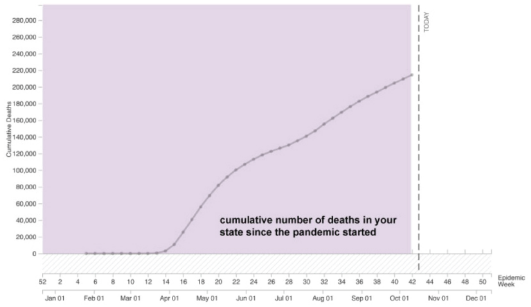

Screen 8

Section 2 Instructions:
Here is how to read these graphs:

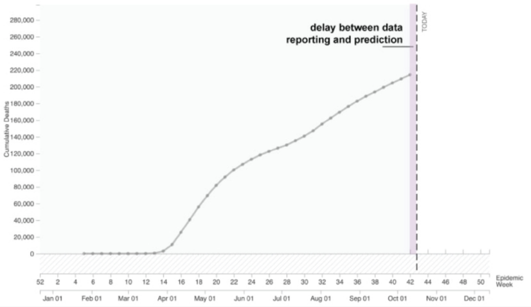

Screen 9

Section 2 Instructions:
Here is how to read these graphs:

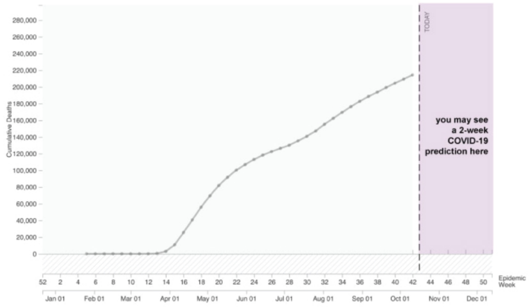

On the next page, you will be asked to view a similar graph for 1.5 mins and describe what you see. A countdown timer will appear on the screen indicating when the minimum 1.5 mins are up, and you may proceed when you are ready. Using the information in the graph, you will be asked to make judgments about COVID-19 for yourself and others.

Screen 10

Please look over this visualization carefully. We would like you to think about this visualization for a minimum of 90 seconds. When the countdown timer is at zero, the "Next" button will appear, and you may proceed to the questions by clicking the button.

Cumulative Number of COVID-19 Deaths in CA

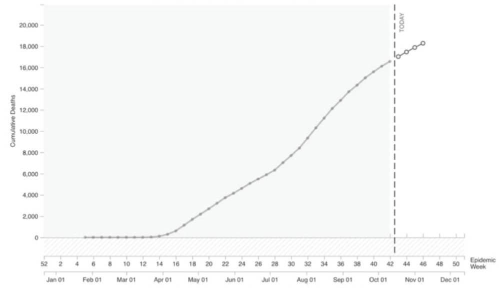

Description: The right of the figure shows a COVID-19 projection.

2.3 COVID-19 knowledge questions from Azlan et al. (2020)

Q	True or False question text	Correct answer
1	The main clinical symptoms of COVID-19 are fever, fatigue, dry cough, and body aches.	True
2	Unlike the common cold, stuffy nose, runny nose, and sneezing are less common in persons infected with the COVID-19 virus.	True
3	There currently is no effective cure for COVID-19, but early symptomatic and supportive treatment can help most patients recover from the infection.	True
4	Not all persons with COVID-19 will develop to severe cases. Only those who are elderly and have chronic illnesses are more likely to be severe cases.	True
5	Eating or touching wild animals would result in the infection by the COVID-19 virus.	False
6	Persons with COVID-19 cannot infect the virus to others if they do not have a fever.	False
7	The COVID-19 virus spreads via respiratory droplets of infected individuals.	True
8	The COVID-19 virus is airborne.	False
9	Ordinary residents can wear face masks to prevent the infection by the COVID-19 virus.	True
10	It is not necessary for children and young adults to take measures to prevent the infection by the COVID-19 virus.	False
11	To prevent the infection by the COVID-19, individuals should avoid going to crowded places and avoid taking public transportation.	True
12	Isolation and treatment of people who are infected with the COVID-19 virus are effective ways to reduce the spread of the virus.	True
13	People who have contact with someone infected with the COVID-19 virus should be immediately isolated in a proper place. In general, the isolation period is 14 days.	True

2.4 Graph literacy questions from Okan et al. (2019)

Question 1 (Correct answer: 25%. 24% and 26% accepted)

Instructions: Please answer the questions below. Do not use a calculator but feel free to use scratch paper.

Here is some information about different forms of cancer.

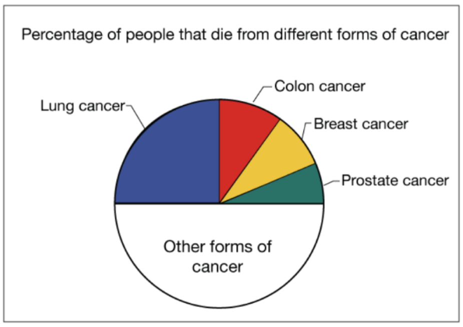

Approximately what percentage of people who die from cancer die from colon cancer, breast cancer, and prostate cancer taken together?
Enter your response in the box below as a percent from 1-100. You don't need to add the percent sign.

Question 2 (Correct answer: 20)

The following figure shows the number of men and women among patients with disease X. The total number of circles is 100.

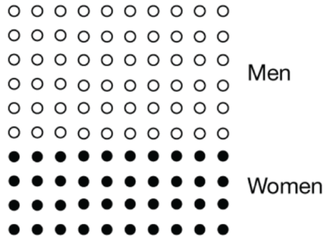

How many more men than women are there among 100 patients with disease X?

Question 3 (Correct answer: They are equal)

In a magazine, you see two advertisements, one on page 5 and another on page 12. Each is for a different drug for treating heart disease, and each includes a graph showing the effectiveness of the drug compared to a placebo (sugar pill).

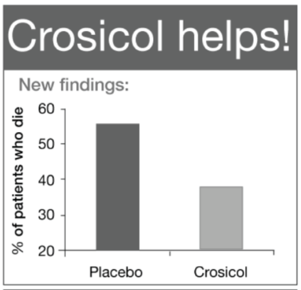

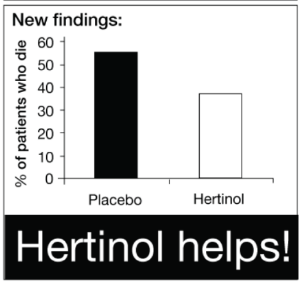

Compared to the placebo, which treatment leads to a larger decrease in the percentage of patients who die?

Crosicol	Hertinol	They are equal	Can not say
○	○	○	○

Question 4 (Correct answer: Can't say)

In the newspaper, you see two advertisements, one on page 15 and another on page 17. Each is for a different treatment of psoriasis, and each includes a graph showing the effectiveness of the treatment over time.

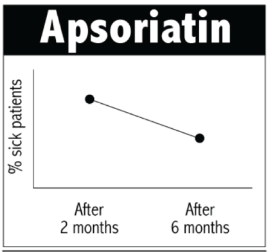

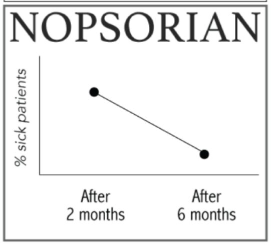

Which of the treatments contributes to a larger decrease in the percentage of sick patients?

Apsoriatin	Nopsorian	They are equal	Can't say
○	○	○	○

2.5 COVID-19 health risk factors (CDC)

These are based on health risk factors produced by the CDC; (https://www.cdc.gov/coronavirus) accessed Feb 26th 2021.

Q	COVID-19 health-risk factors
1	Cancer
2	Chronic kidney disease
3	COPD (Chronic obstructive pulmonary disease)
4	Immunocompromised state (weakened immune system) from solid organ transplant
5	Obesity (Body Mass Index [BMI] of 30 or higher)
6	Serious heart conditions such as heart failure, coronary artery disease, or cardiomyopathies
7	Sickle cell disease
8	Type 2 diabetes mellitus
9	Asthma
10	Cerebrovascular disease (affects blood vessels and blood supply to the brain)
11	Cystic fibrosis
12	Hypertension or high blood pressure
13	Immunocompromised state (weakened immune system) from blood/bone marrow transplant, immune deficiencies, HIV, use of corticosteroids, or other immune weakening medicines
14	Neurologic conditions, such as dementia
15	Liver disease
16	Pulmonary fibrosis (having damaged or scarred lung tissues
17	Thalassemia (a type of blood disorder)
18	Type 1 diabetes mellitus
19	Pregnancy
20	Smoking
21	Prefer not to say

3 Stimuli

3.1 Exp 1

No forecast

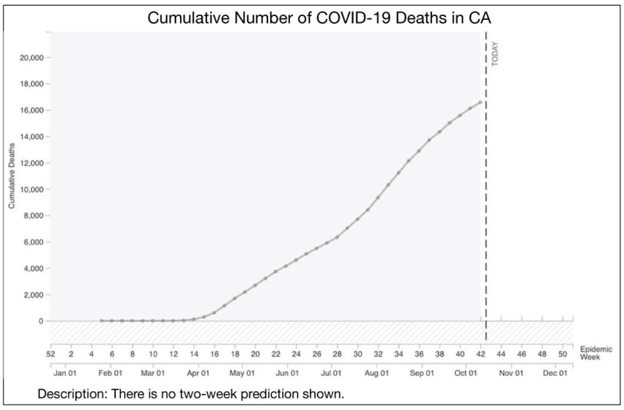

Mean

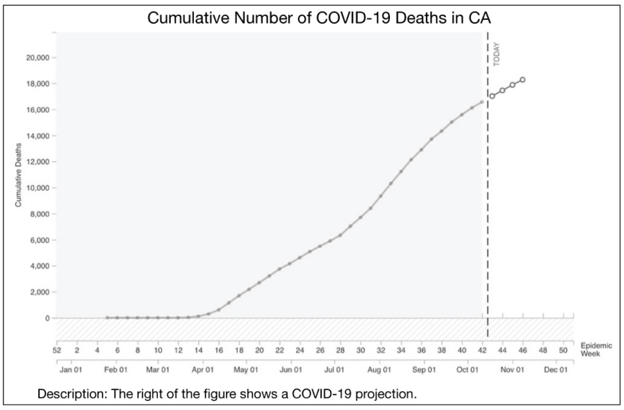

Mean + 50%CI

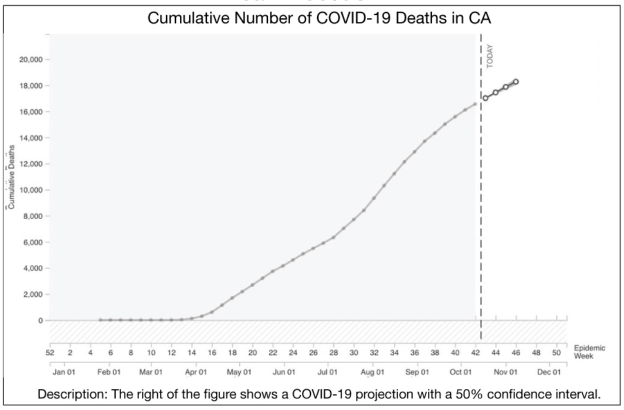

3 Models Spaning 50%CIs

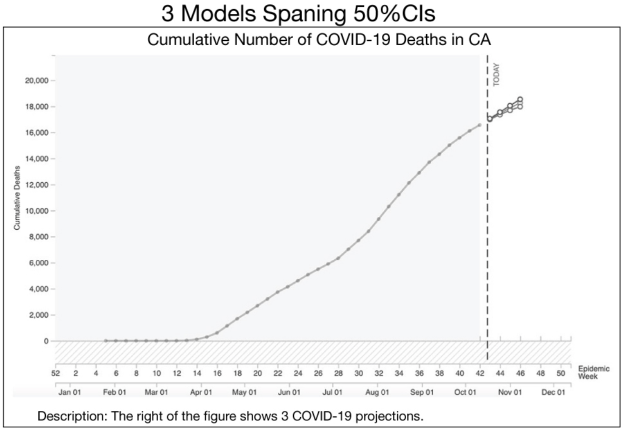

Mean + 95%CI

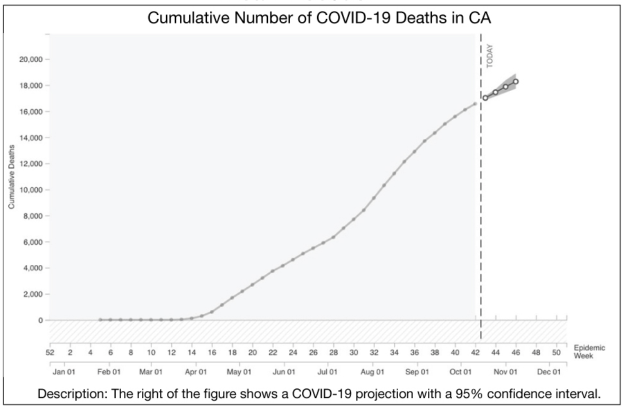

3 Models Spaning 95%CIs

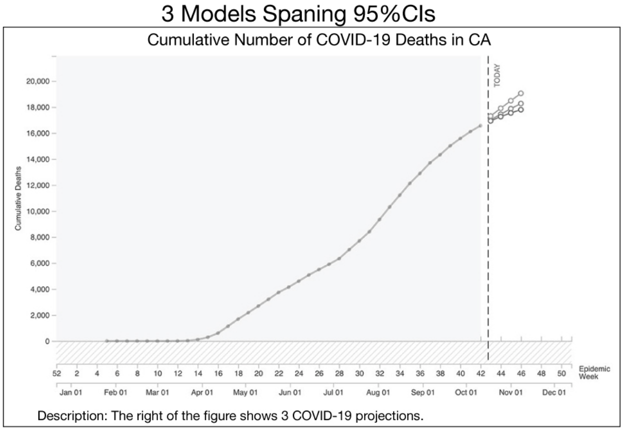

6 Models

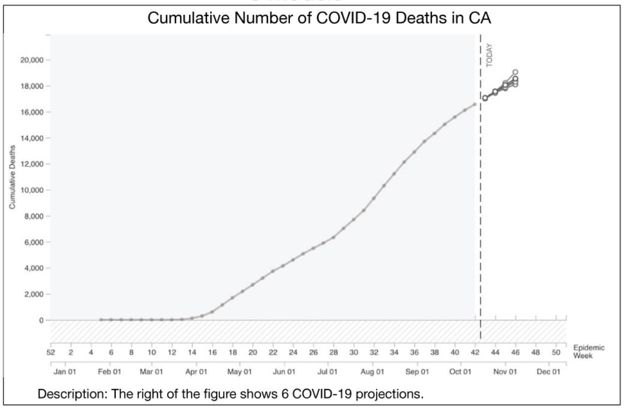

All Models

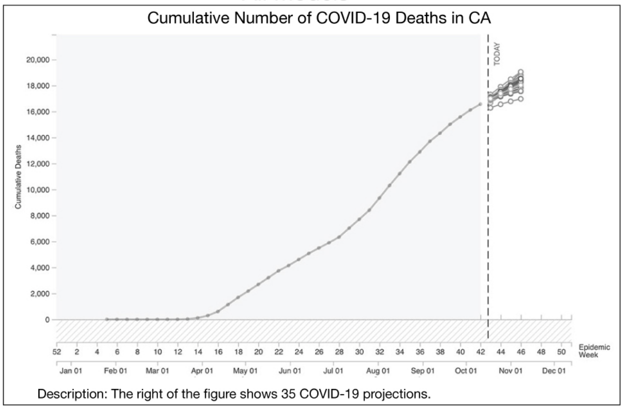

No forecast

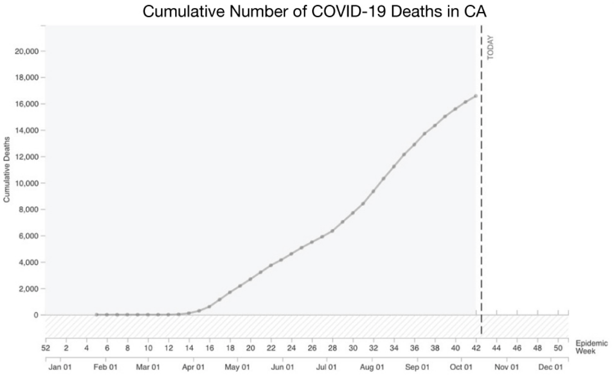

Description: There is no two-week prediction shown.

Mean

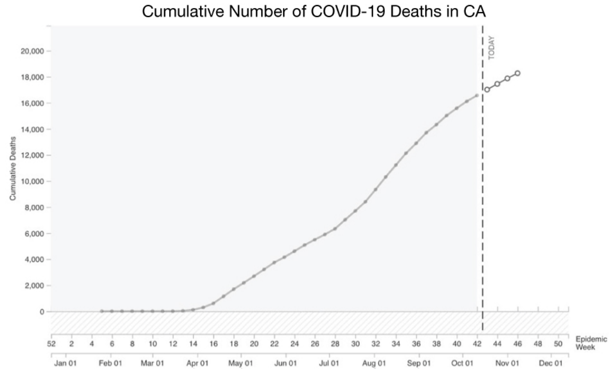

Description: The right of the figure shows a COVID-19 projection.

Mean + 50%CI

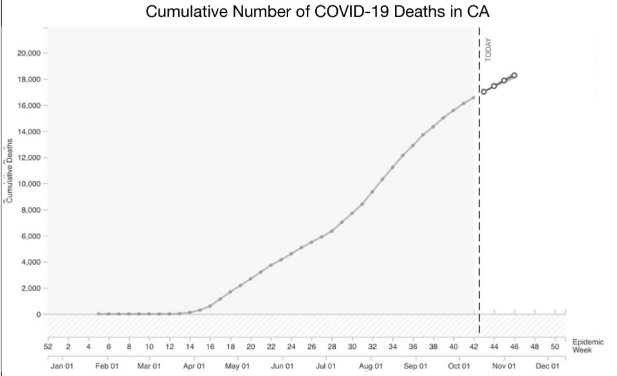

Description: The right of the figure shows a COVID-19 projection with a 50% confidence interval.

3 Models Spaning 50%CIs

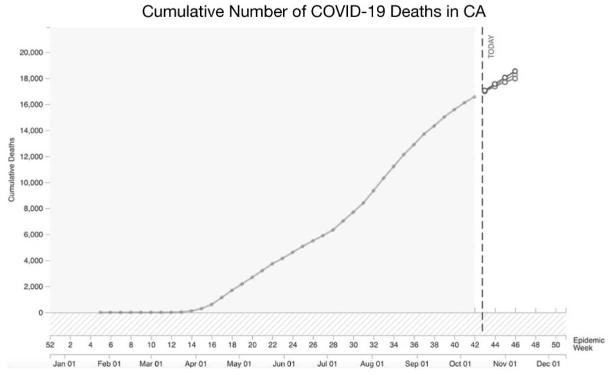

Description: The right of the figure shows 3 COVID-19 projections.

Mean + 95%CI

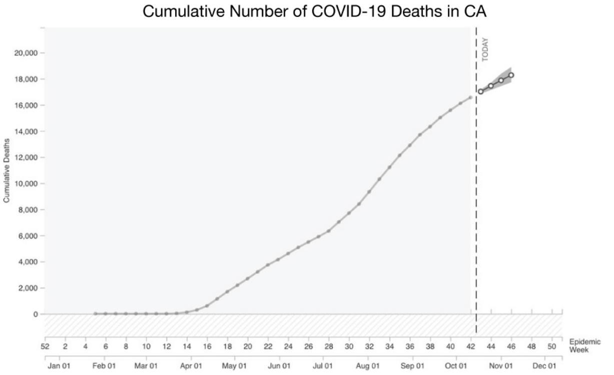

Description: The right of the figure shows a COVID-19 projection with a 95% confidence interval.

3 Models Spaning 95%CIs

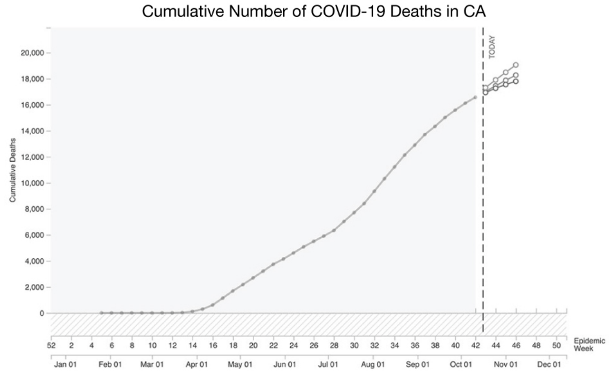

Description: The right of the figure shows 3 COVID-19 projections.

6 Models

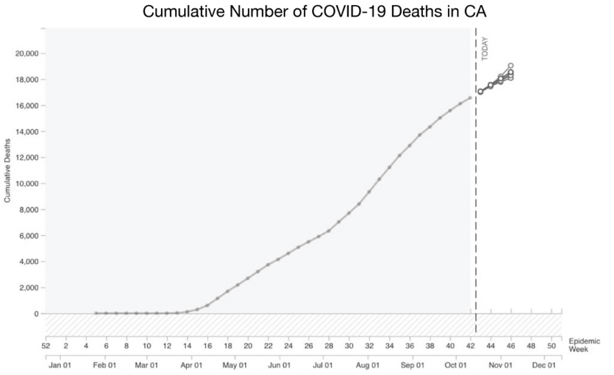

Description: The right of the figure shows 6 COVID-19 projections.

All Models

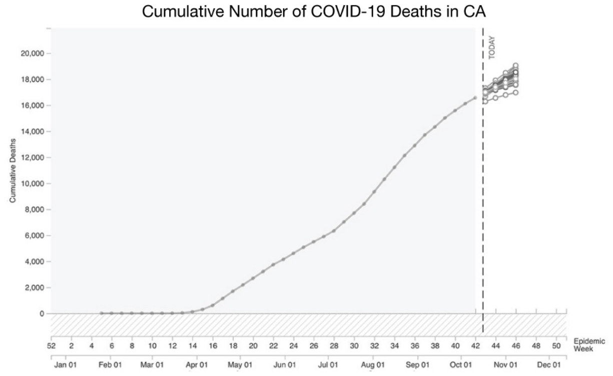

Description: The right of the figure shows 35 COVID-19 projections.

3.2 Exp 2

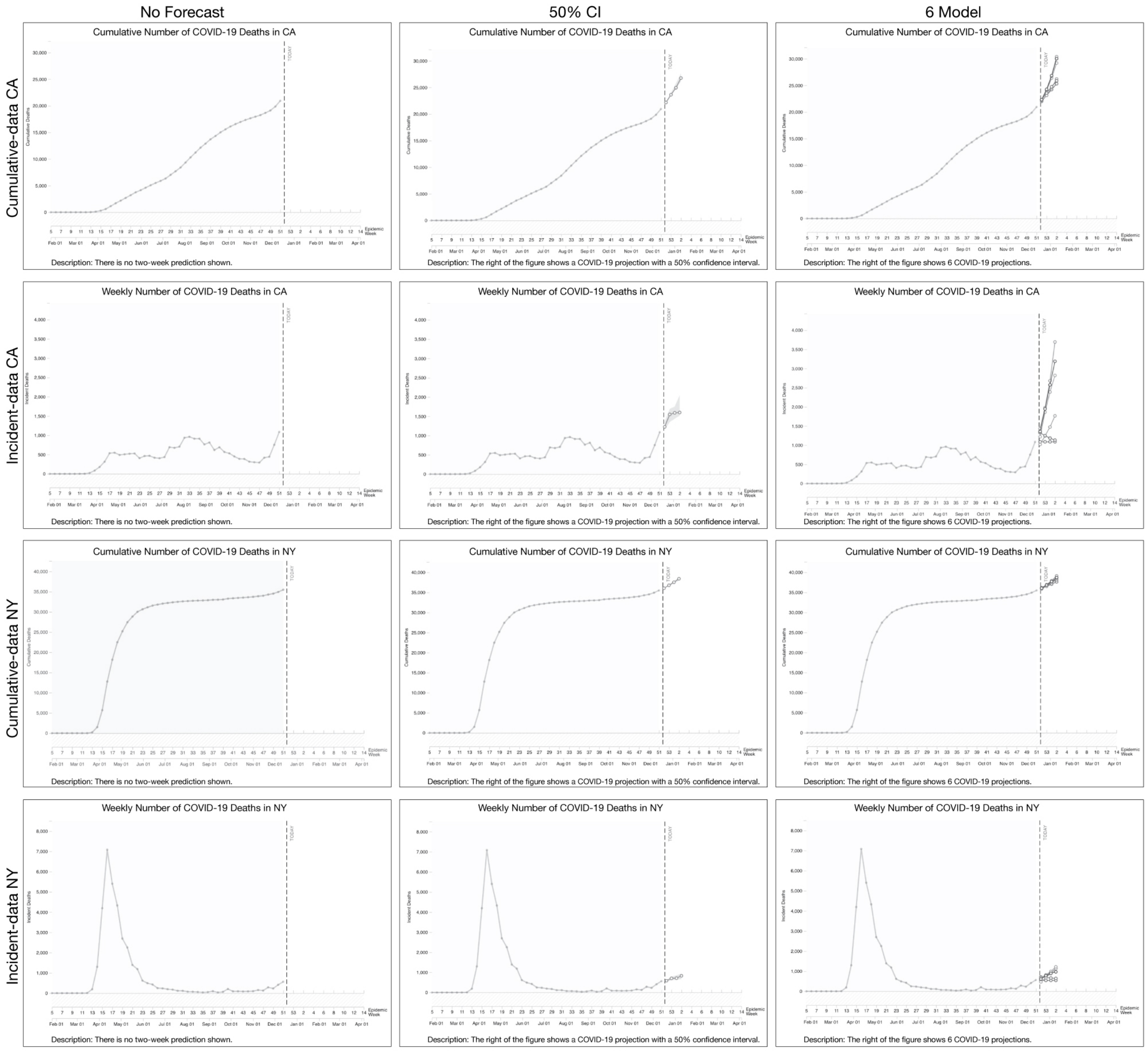
No Forecast
50% CI
6 Model
Cumulative-data CA
Incident-data CA
Cumulative-data NY
Incident-data NY
Cumulative Number of COVID-19 Deaths in CA
Weekly Number of COVID-19 Deaths in CA
Cumulative Number of COVID-19 Deaths in NY
Weekly Number of COVID-19 Deaths in NY
Description: There is no two-week prediction shown.
Description: The right of the figure shows a COVID-19 projection with a 50% confidence interval.
Description: The right of the figure shows 6 COVID-19 projections.

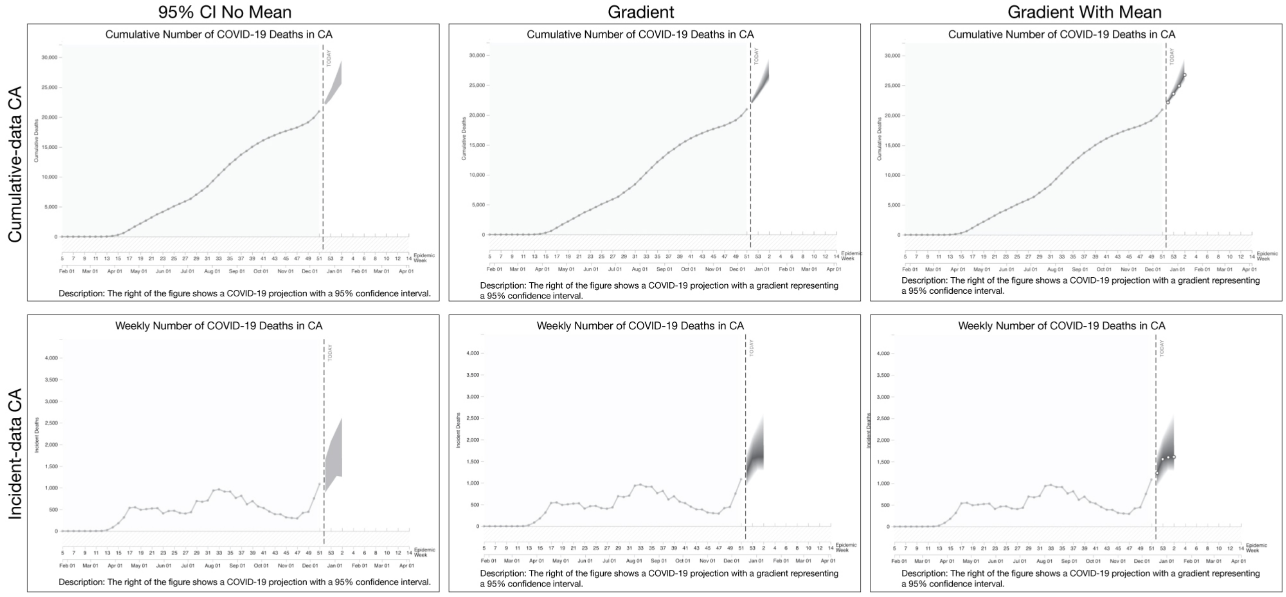
95% CI No Mean
Gradient
Gradient With Mean
Cumulative-data CA
Incident-data CA
Cumulative Number of COVID-19 Deaths in CA
Weekly Number of COVID-19 Deaths in CA
Description: The right of the figure shows a COVID-19 projection with a 95% confidence interval.
Description: The right of the figure shows a COVID-19 projection with a gradient representing a 95% confidence interval.

4 Analysis

4.1 Exp 1

4.1.1 Model 1 Omnibus

The following results used multi-level models in which risk estimates (1-7) were specified as the outcome variable (see R notation with descriptions of variable transformations below).

Risk estimate (outcome) ~
Time point * Visualization (fixed-interaction) +
Time point * Y-axis (fixed-interaction) +
Y-axis * Visualization (fixed-interaction) +
Time point (fixed-effect, categorical 2 levels, referent = pre-visualization exposure) +
Visualization (fixed-effect, categorical 8 levels, referent = No Forecast) +
Y-axis (fixed-effect, categorical 2 levels, referent = cumulative) +
Question type (fixed-effect, numeric, coded as -1, -.5, .5, 1) +
Target type (fixed-effect, categorical 3 levels, referent = self) +
Graph literacy centered centered (fixed-effect, numeric 0-4, centered on grand mean) +
COVID-19-knowlege centered (fixed-effect, numeric 1-13, centered on grand mean) +
COVID-19-health-risk centered (fixed-effect, numeric 1-20, centered on grand mean) +
Contracted COVID-19 centered (fixed-effect, dichotomous, yes = 1 no = -1) +
Tested for COVID-19 centered (fixed-effect, dichotomous, yes = 1 no = -1) +
Age centered (fixed-effect, numeric, centered on grand mean)+
Gender centered (fixed-effect, dichotomous, male = 1 female = -1) +
Education centered (fixed-effect, numeric 1-8, centered on grand mean) +
(Target type + Question type + Time point | Participant Id)

	Estimate	Std..Error	t.value	p.z	2.5 %	97.5 %
(Intercept)	4.728	0.101	46.977	0.000	4.530	4.925
Time point	0.294	0.047	6.297	0.000	0.203	0.386
Y-axis	-0.094	0.137	-0.687	0.492	-0.363	0.175
Visualization: Mean	0.169	0.137	1.232	0.218	-0.100	0.438
Visualization: CI 50	0.184	0.137	1.345	0.179	-0.084	0.453
Visualization: 3 Models CI 50	0.199	0.138	1.447	0.148	-0.070	0.469
Visualization: CI 95	0.269	0.138	1.950	0.051	-0.001	0.539
Visualization: 3 Models CI 95	0.224	0.137	1.635	0.102	-0.045	0.492
Visualization: 6 Models	0.176	0.138	1.275	0.202	-0.094	0.446
Visualization: All Models	0.274	0.138	1.989	0.047	0.004	0.544
Question type	0.195	0.015	12.975	0.000	0.166	0.225
Target type: Self	-1.382	0.028	-48.656	0.000	-1.438	-1.326
Target type: Young	-1.004	0.024	-42.170	0.000	-1.050	-0.957
Graph Literacy (Centered)	-0.176	0.022	-7.955	0.000	-0.219	-0.132
COVID-19 Knowledge (Centered)	-0.045	0.013	-3.332	0.001	-0.071	-0.018
Health Risk Factors (Centered)	0.070	0.021	3.365	0.001	0.029	0.110
Contracted COVID-19 (Centered)	0.163	0.035	4.605	0.000	0.094	0.232
Tested for COVID-19 (Centered)	0.071	0.027	2.636	0.008	0.018	0.123
Age (Centered)	-0.001	0.002	-0.358	0.720	-0.005	0.004
Gender (Centered)	-0.015	0.024	-0.599	0.549	-0.063	0.033
Education (Centered)	0.034	0.015	2.267	0.023	0.005	0.063
**Time point * Y-axis**	**-0.326**	**0.031**	**-10.476**	**0.000**	**-0.388**	**-0.265**
:Time point * Visualization: Mean	-0.097	0.062	-1.561	0.119	-0.220	0.025
**Time point * Visualization: CI 50**	**-0.163**	**0.062**	**-2.622**	**0.009**	**-0.285**	**-0.041**
Time point * Visualization: 3 Models CI 50	-0.116	0.062	-1.855	0.064	-0.238	0.007
Time point * Visualization: CI 95	-0.036	0.062	-0.573	0.567	-0.157	0.086
Time point * Visualization: 3 Models CI 95	-0.042	0.062	-0.671	0.502	-0.164	0.080
Time point * Visualization: 6 Models	-0.006	0.062	-0.093	0.926	-0.128	0.117
Time point * Visualization: All Models	-0.011	0.063	-0.178	0.858	-0.134	0.112
Y-axis * Visualization: Mean	0.100	0.194	0.518	0.605	-0.280	0.481
Y-axis * Visualization: CI 50	-0.023	0.194	-0.117	0.907	-0.403	0.357
Y-axis * Visualization: 3 Models CI 50	-0.059	0.194	-0.305	0.760	-0.439	0.320
Y-axis * Visualization: CI 95	-0.333	0.195	-1.712	0.087	-0.714	0.048
Y-axis * Visualization: 3 Models CI 95	0.096	0.194	0.494	0.621	-0.284	0.475
Y-axis * Visualization: 6 Models	0.017	0.194	0.090	0.929	-0.363	0.398
Y-axis * Visualization: All Models	-0.078	0.195	-0.402	0.688	-0.460	0.303

Effect size for the model:

```
##              R2m       R2c
## [1,] 0.2021439 0.5675612
```

The analysis revealed an interaction between time point and y-axis (b = -.33, p = .000, CIs[-.39, -.27]) (Table 4.1.1 highlighted in blue). The second interaction revealed by the main analysis was between time points and the No Forecast vs. CI 50 (shown in Table 4.1.1, highlighted in green) (b =-.16, SD = .06, p = .000, CIs[-.29, -.04]).

4.1.1.1 Mean risk judgments for each condition

Table showing summary statics for each condition

Visualization	Y-axis	Time-point	N	Mean Judgment	sd	se	ci
3 Models CI 50	Cumulative	Pre	900	4.073	1.954	0.065	0.128
3 Models CI 50	Cumulative	Post	900	4.224	1.857	0.062	0.121
3 Models CI 50	Incident	Pre	900	3.903	2.014	0.067	0.132
3 Models CI 50	Incident	Post	900	3.756	1.782	0.059	0.117
3 Models CI 95	Cumulative	Pre	900	4.079	1.941	0.065	0.127
3 Models CI 95	Cumulative	Post	900	4.281	1.826	0.061	0.119
3 Models CI 95	Incident	Pre	900	4.139	1.837	0.061	0.120
3 Models CI 95	Incident	Post	900	4.110	1.654	0.055	0.108
6 Models	Cumulative	Pre	900	4.043	1.918	0.064	0.125
6 Models	Cumulative	Post	900	4.283	1.753	0.058	0.115
6 Models	Incident	Pre	900	3.881	2.028	0.068	0.133
6 Models	Incident	Post	900	3.902	1.879	0.063	0.123
All Models	Cumulative	Pre	900	4.018	1.952	0.065	0.128
All Models	Cumulative	Post	900	4.332	1.830	0.061	0.120
All Models	Incident	Pre	900	3.851	2.075	0.069	0.136
All Models	Incident	Post	900	3.800	1.943	0.065	0.127
CI 50	Cumulative	Pre	900	4.081	1.936	0.065	0.127
CI 50	Cumulative	Post	900	4.242	1.868	0.062	0.122
CI 50	Incident	Pre	900	3.852	1.997	0.067	0.131
CI 50	Incident	Post	900	3.632	1.752	0.058	0.115
CI 95	Cumulative	Pre	900	4.314	1.757	0.059	0.115
CI 95	Cumulative	Post	900	4.569	1.646	0.055	0.108
CI 95	Incident	Pre	888	3.681	1.891	0.063	0.125
CI 95	Incident	Post	888	3.604	1.738	0.058	0.114
Mean	Cumulative	Pre	900	3.941	1.970	0.066	0.129
Mean	Cumulative	Post	900	4.124	1.834	0.061	0.120
Mean	Incident	Pre	900	3.889	2.142	0.071	0.140
Mean	Incident	Post	900	3.774	1.888	0.063	0.123
No Forecast	Cumulative	Pre	900	3.767	1.998	0.067	0.131
No Forecast	Cumulative	Post	900	4.106	1.841	0.061	0.120
No Forecast	Incident	Pre	900	3.706	1.970	0.066	0.129
No Forecast	Incident	Post	900	3.623	1.798	0.060	0.118

4.1.2 Plot of primary task results

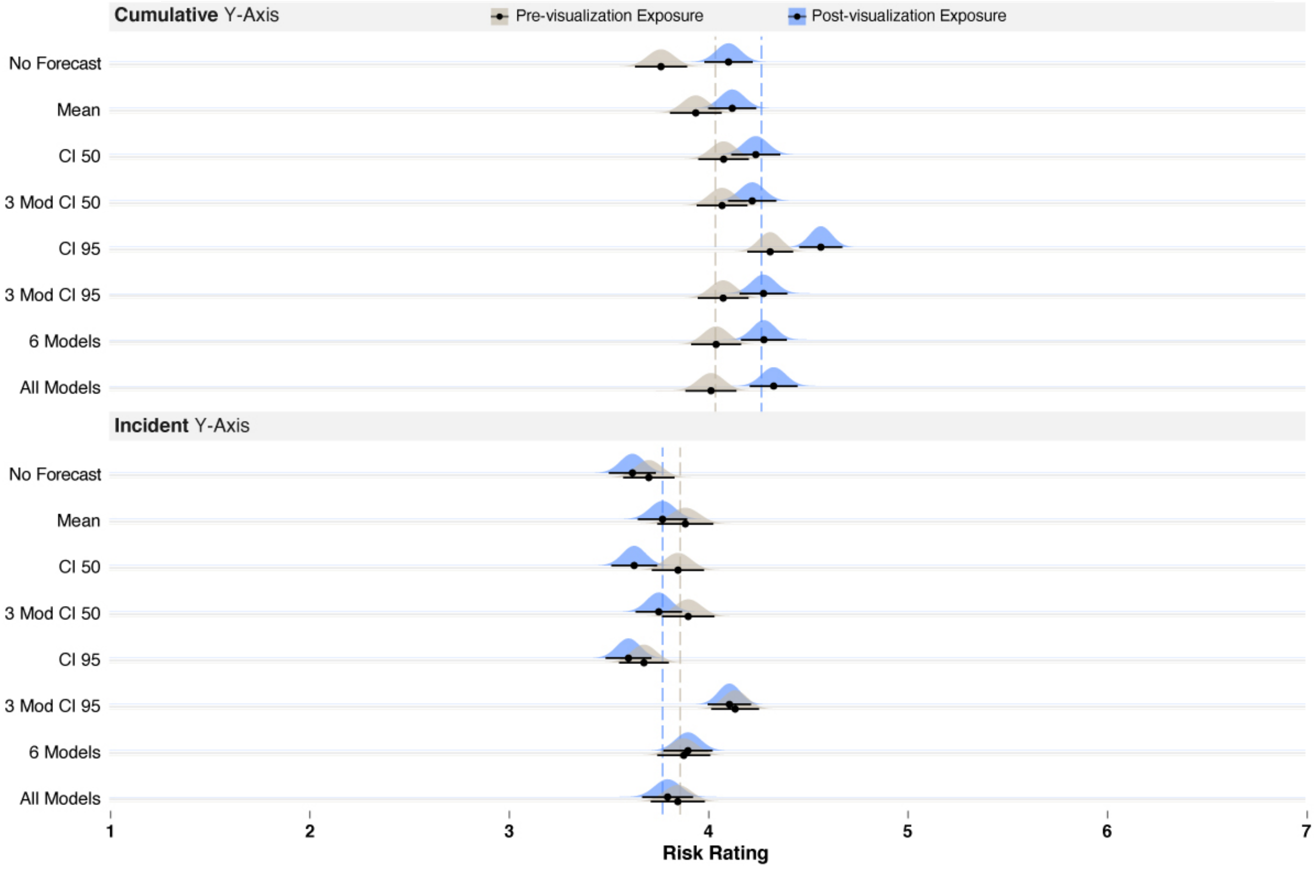

Results of Experiment 1, where pre-visualization risk judgments are colored gray and post-visualization judgments blue, for cumulative y-axis (top) and incident y-axis (bottom). Dashed lines show the mean pre- and post-visualization risk judgments for each y-axis group as a whole. Black bars show 95% confidence intervals around the mean (black dot) for each condition using the Cousineau-Morey method and the density plots were generated from this data.

4.1.3 Interaction analysis: Time point * Y-axis

To break down the interaction between time point and y-axis we used the same modeling procedure described above and conducted follow-up analyses with only the cumulative (Table 4.1.3.1) or the incident data (Table 4.1.3.2).

4.1.3.1 Model using cumulative data and No Forecast as the referent This model revealed that the cumulative-data evoked a significant increase in risk estimates (means shown in the dashed lines of Figure 4.1.2, top) (Table below highlighted in green) ($b = .34$, $SD = .06$, $p = .000$, $CIs[.21, .46]$).

	Estimate	Std..Error	t.value	p.z	2.5 %	97.5 %
(Intercept)	4.762	0.106	44.732	0.000	4.554	4.971
**Time point**	**0.336**	**0.062**	**5.379**	**0.000**	**0.213**	**0.458**
Visualization: All Models	0.261	0.141	1.844	0.065	-0.016	0.537
Visualization: 6 Models	0.158	0.141	1.118	0.264	-0.119	0.435
Visualization: 3 Models CI 95	0.285	0.141	2.018	0.044	0.008	0.561
Visualization: 3 Models CI 50	0.173	0.141	1.223	0.221	-0.104	0.450
Visualization: CI 95	0.245	0.143	1.719	0.086	-0.034	0.525
Visualization: CI 50	0.169	0.141	1.199	0.231	-0.107	0.445
Visualization: Mean	0.170	0.141	1.208	0.227	-0.106	0.446
Question type	0.161	0.021	7.546	0.000	0.119	0.203
Target type: Self	-1.353	0.042	-32.473	0.000	-1.434	-1.271
Target type: Young	-0.972	0.035	-27.928	0.000	-1.040	-0.904
Graph Literacy (Centered)	-0.209	0.032	-6.476	0.000	-0.273	-0.146
COVID-19 Knowledge (Centered)	-0.041	0.020	-2.036	0.042	-0.080	-0.002
Contracted COVID-19 (Centered)	0.198	0.048	4.101	0.000	0.103	0.293
Tested for COVID-19 (Centered)	0.123	0.039	3.153	0.002	0.047	0.200
Health Risk Factors (Centered)	0.025	0.028	0.897	0.370	-0.030	0.080
Age (Centered)	0.001	0.003	0.220	0.826	-0.006	0.007
Gender (Centered)	-0.084	0.035	-2.374	0.018	-0.153	-0.015
Education (Centered)	0.057	0.022	2.672	0.008	0.015	0.100
Time point *Visualization: All Models	-0.024	0.089	-0.270	0.787	-0.199	0.151
Time point *Visualization: 6 Models	-0.097	0.089	-1.084	0.278	-0.271	0.078
Time point *Visualization: 3 Models CI 95	-0.134	0.088	-1.519	0.129	-0.307	0.039
Time point *Visualization: 3 Models CI 50	-0.172	0.089	-1.940	0.052	-0.347	0.002
Time point *Visualization: CI 95	-0.073	0.088	-0.829	0.407	-0.246	0.100
Time point *Visualization: CI 50	-0.177	0.089	-2.000	0.046	-0.351	-0.004
Time point *Visualization: Mean	-0.159	0.089	-1.794	0.073	-0.333	0.015

4.1.3.2 Model using incident data and No Forecast as the referent In contrast, the incident data did not show a significant change in participants' evaluations of risk (means shown in the dashed lines of Figure 4.1.2, bottom) (Table below highlighted in green) (b = -.06, SD = .06, p = .33, CIs[-.18, .06]). These findings suggest that participants increased their risk estimates by a third of a point on a 7 point Likert scale after viewing the cumulative-data but not the visualizations that used the incident COVID-19 death data.

	Estimate	Std..Error	t.value	p.z	2.5 %	97.5 %
(Intercept)	4.601	0.101	45.452	0.000	4.402	4.799
**Time point**	**-0.061**	**0.062**	**-0.985**	**0.325**	**-0.183**	**0.060**
Visualization: All Models	0.182	0.134	1.362	0.173	-0.080	0.445
Visualization: 6 Models	0.183	0.133	1.381	0.167	-0.077	0.444
Visualization: 3 Models CI 95	0.321	0.133	2.415	0.016	0.061	0.582
Visualization: 3 Models CI 50	0.147	0.133	1.105	0.269	-0.114	0.408
Visualization: CI 95	-0.062	0.133	-0.466	0.641	-0.323	0.199
Visualization: CI 50	0.165	0.133	1.247	0.212	-0.095	0.425
Visualization: Mean	0.246	0.133	1.848	0.065	-0.015	0.506
Question type	0.229	0.021	10.869	0.000	0.188	0.270
Target type: Self	-1.412	0.039	-36.573	0.000	-1.487	-1.336
Target type: Young	-1.035	0.032	-31.932	0.000	-1.099	-0.972
Graph Literacy (Centered)	-0.153	0.030	-5.164	0.000	-0.211	-0.095
COVID-19 Knowledge (Centered)	-0.038	0.018	-2.138	0.032	-0.073	-0.003
Contracted COVID-19 (Centered)	0.117	0.051	2.291	0.022	0.017	0.217
Tested for COVID-19 (Centered)	0.020	0.036	0.539	0.590	-0.051	0.090
Health Risk Factors (Centered)	0.112	0.030	3.675	0.000	0.052	0.172
Age (Centered)	-0.002	0.003	-0.673	0.501	-0.008	0.004
Gender (Centered)	0.043	0.033	1.285	0.199	-0.022	0.108
Education (Centered)	0.007	0.020	0.326	0.745	-0.033	0.047
Time point *Visualization: All Models	-0.014	0.088	-0.165	0.869	-0.186	0.157
Time point *Visualization: 6 Models	0.073	0.087	0.836	0.403	-0.098	0.243
Time point *Visualization: 3 Models CI 95	0.039	0.087	0.450	0.653	-0.131	0.210
Time point *Visualization: 3 Models CI 50	-0.072	0.087	-0.832	0.405	-0.243	0.098
Time point *Visualization: CI 95	-0.019	0.087	-0.223	0.823	-0.190	0.151
Time point *Visualization: CI 50	-0.163	0.087	-1.876	0.061	-0.332	0.007
Time point *Visualization: Mean	-0.052	0.087	-0.599	0.549	-0.222	0.118

4.1.4 Interaction analysis: Time point * No Forecast vs. CI 50

To break down this interaction between time points and the No Forecast vs. CI 50 visualizations (shown in Table 4.1.1, highlighted in green) we ran separate models for the No Forecast (Table 4.1.4.1) and CI 50 visualizations (Table 4.1.4.2).

4.1.4.1 Model with only participants in the No Forecast group These analysis revealed a larger increase in risk estimates when participants viewed the COVID-19 line chart with No forecast (Table

below highlighted in green) (b = .33, SD = .07, p = .000, CIs[.20, .47]; pre-visualization exposure m = 3.73, SD = 1.99, post-visualization exposure m = 3.86, SD = 1.83, change = .13) compared to the visualizations showing 50% confidence intervals.

	Estimate	Std..Error	t.value	p.z	2.5 %	97.5 %
(Intercept)	4.498	0.120	37.471	0.000	4.262	4.733
**Time point**	**0.330**	**0.069**	**4.787**	**0.000**	**0.195**	**0.465**
Y-axis	-0.003	0.125	-0.027	0.978	-0.248	0.241
Question type	0.301	0.041	7.309	0.000	0.220	0.382
Target type: Self	-1.344	0.075	-18.021	0.000	-1.491	-1.198
Target type: Young	-0.942	0.068	-13.813	0.000	-1.076	-0.808
Graph Literacy (Centered)	-0.249	0.055	-4.491	0.000	-0.358	-0.140
COVID-19 Knowledge (Centered)	-0.028	0.032	-0.881	0.378	-0.090	0.034
Contracted COVID-19 (Centered)	-0.055	0.093	-0.599	0.549	-0.237	0.126
Tested for COVID-19 (Centered)	0.079	0.067	1.189	0.235	-0.052	0.211
Health Risk Factors (Centered)	0.213	0.068	3.131	0.002	0.080	0.346
Age (Centered)	-0.017	0.006	-2.625	0.009	-0.030	-0.004
Gender (Centered)	-0.128	0.063	-2.029	0.042	-0.251	-0.004
Education (Centered)	0.021	0.037	0.577	0.564	-0.051	0.094
Time point *Y-axis	-0.392	0.098	-3.994	0.000	-0.584	-0.199

4.1.4.2 Model with only participants in the CI50 group This model showed a smaller effect of time point (Table below highlighted in green) (b = .17, SD = .06, p = .008, CIs[.05, .3]; pre-visualization exposure m = 3.97, SD = 1.97, post-visualization exposure m = 3.94, SD = 1.82, change = .03). These results suggest that the interaction was driven by the more prominent increase in risk estimates when participants viewed visualizations with No forecast compared to visualizations that showed future forecasts with 50% confidence intervals.

	Estimate	Std..Error	t.value	p.z	2.5 %	97.5 %
(Intercept)	4.962	0.119	41.679	0.000	4.729	5.195
**Time point**	**0.171**	**0.064**	**2.655**	**0.008**	**0.045**	**0.298**
Y-axis	-0.036	0.135	-0.269	0.788	-0.302	0.229
Question type	0.147	0.038	3.831	0.000	0.072	0.221
Target type: Self	-1.434	0.077	-18.674	0.000	-1.584	-1.283
Target type: Young	-1.072	0.070	-15.327	0.000	-1.209	-0.935
Graph Literacy (Centered)	-0.181	0.056	-3.227	0.001	-0.291	-0.071
COVID-19 Knowledge (Centered)	-0.039	0.035	-1.106	0.269	-0.108	0.030
Contracted COVID-19 (Centered)	0.275	0.097	2.840	0.005	0.085	0.464
Tested for COVID-19 (Centered)	0.061	0.072	0.854	0.393	-0.079	0.201
Health Risk Factors (Centered)	0.115	0.060	1.920	0.055	-0.002	0.233
Age (Centered)	0.007	0.007	1.026	0.305	-0.006	0.020
Gender (Centered)	-0.059	0.066	-0.901	0.367	-0.188	0.069
Education (Centered)	0.097	0.038	2.533	0.011	0.022	0.172
Time point *Y-axis	-0.405	0.090	-4.482	0.000	-0.582	-0.228

4.1.5 Post hoc test: Full model CI 50 as the referent

As a post hoc analysis, we tested if CI 50 showed less of a change in risk estimates compared to the other visualizations, by running the previously described omnibus model with CI 50 as the referent. Note that the model failed to converge, and we removed time point from the random effects structure but left it in as a fixed effect to support convergence.

Risk estimate (outcome) ∼
Time point * Visualization (fixed-interaction) +
Time point * Y-axis (fixed-interaction) +
Y-axis * Visualization (fixed-interaction) +
Time point (fixed-effect, categorical 2 levels, referent = pre-visualization exposure) +
Visualization (fixed-effect, categorical 8 levels, referent = No Forecast) +
Y-axis (fixed-effect, categorical 2 levels, referent = cumulative) +
Question type (fixed-effect, numeric, coded as -1, -.5, .5, 1) +
Target type (fixed-effect, categorical 3 levels, referent = self) +
Graph-literacy centered centered (fixed-effect, numeric 0-4, centered on grand mean) +
COVID-19-knowlege centered (fixed-effect, numeric 1-13, centered on grand mean) +
COVID-19-health-risk centered (fixed-effect, numeric 1-20, centered on grand mean) +
Contracted COVID-19 centered (fixed-effect, dichotomous, yes = 1 no = -1) +
Tested for COVID-19 centered (fixed-effect, dichotomous, yes = 1 no = -1) +
Age centered (fixed-effect, numeric, centered on grand mean)+

Gender centered (fixed-effect, dichotomous, male = 1 female = -1) +
Education centered (fixed-effect, numeric 1-8, centered on grand mean) +
(Target type + Question type | Participant Id)

This model (Table below) revealed multiple interactions between time point and CI 50 vs. All Models, 6 Models, CI 95, 3 Models CI 95, and the previously described interaction with No Forecast (highlighted in blue).

	Estimate	Std..Error	t.value	p.z	2.5 %	97.5 %
(Intercept)	4.922	0.104	47.542	0.000	4.720	5.125
Time point	0.128	0.039	3.274	0.001	0.051	0.204
Y-axis	-0.143	0.140	-1.024	0.306	-0.417	0.131
Visualization: No Forecast	-0.188	0.142	-1.330	0.184	-0.466	0.089
Visualization: Mean	-0.035	0.142	-0.247	0.805	-0.314	0.243
Visualization: 3 Models CI 50	0.003	0.142	0.022	0.982	-0.276	0.282
Visualization: CI 95	0.085	0.142	0.596	0.551	-0.193	0.363
Visualization: 3 Models CI 95	0.019	0.141	0.136	0.892	-0.257	0.296
Visualization: All Models	0.084	0.143	0.587	0.557	-0.196	0.364
Visualization: 6 Models	-0.030	0.142	-0.211	0.833	-0.309	0.249
Question type	0.195	0.015	12.975	0.000	0.166	0.225
Target type: Self	-1.382	0.028	-48.655	0.000	-1.438	-1.326
Target type: Young	-1.004	0.024	-42.170	0.000	-1.050	-0.957
Graph Literacy (Centered)	-0.180	0.022	-8.002	0.000	-0.224	-0.136
COVID-19 Knowledge (Centered)	-0.043	0.014	-3.113	0.002	-0.070	-0.016
Health Risk Factors (Centered)	0.075	0.021	3.550	0.000	0.034	0.116
Contracted COVID-19 (Centered)	0.162	0.036	4.508	0.000	0.092	0.233
Tested for COVID-19 (Centered)	0.066	0.027	2.396	0.017	0.012	0.119
Age (Centered)	-0.002	0.002	-0.741	0.459	-0.006	0.003
Gender (Centered)	-0.013	0.025	-0.535	0.593	-0.062	0.036
Education (Centered)	0.033	0.015	2.178	0.029	0.003	0.063
**Time point *Y-axis**	**-0.318**	**0.026**	**-12.180**	**0.000**	**-0.369**	**-0.267**
**Time point *Visualization: No Forecast**	**0.166**	**0.052**	**3.182**	**0.001**	**0.064**	**0.268**
Time point *Visualization: Mean	0.073	0.052	1.406	0.160	-0.029	0.175
Time point *Visualization: 3 Models CI 50	0.045	0.052	0.873	0.383	-0.057	0.148
**Time point *Visualization: CI 95**	**0.120**	**0.052**	**2.306**	**0.021**	**0.018**	**0.222**
**Time point *Visualization: 3 Models CI 95**	**0.112**	**0.052**	**2.155**	**0.031**	**0.010**	**0.214**
**Time point *Visualization: All Models**	**0.158**	**0.052**	**3.017**	**0.003**	**0.055**	**0.261**
**Time point *Visualization: 6 Models**	**0.153**	**0.052**	**2.938**	**0.003**	**0.051**	**0.256**
Y-axis * Visualization: No Forecast	0.026	0.198	0.130	0.897	-0.362	0.413
Y-axis * Visualization: Mean	0.156	0.197	0.792	0.428	-0.230	0.542
Y-axis * Visualization: 3 Models CI 50	-0.009	0.197	-0.044	0.965	-0.394	0.377
Y-axis * Visualization: CI 95	-0.301	0.196	-1.530	0.126	-0.686	0.085
Y-axis * Visualization: 3 Models CI 95	0.170	0.196	0.864	0.388	-0.215	0.554
Y-axis * Visualization: All Models	-0.051	0.198	-0.257	0.797	-0.439	0.337
Y-axis * Visualization: 6 Models	0.088	0.197	0.443	0.657	-0.299	0.474

Effect size for the model:

```
##            R2m       R2c
## [1,] 0.2022976 0.5575231
```

4.1.5.1 Interaction analysis: Time point * CI50 and All Models, 6 Models, CI95, and 3 Models CI95

To breakdown these interactions, we ran separate models for All Models (Table 4.1.5.2), 6-model (Table 4.1.5.3), CI 95 (Table 4.1.5.4), and 3 models CI95 (Table 4.1.5.5). All of the visualizations that had interactions with CI 50 showed a significant increase in risk estimates (highlighted in green in each table).

4.1.5.2 Model with only participants in the All Models group

This model had a significant effect of time point highlighted in green below.

	Estimate	Std..Error	t.value	p.z	2.5 %	97.5 %
(Intercept)	5.090	0.124	41.054	0.000	4.847	5.333
**Time point**	**0.314**	**0.055**	**5.688**	**0.000**	**0.206**	**0.422**
Y-axis	-0.120	0.133	-0.898	0.369	-0.381	0.141
Question type	0.238	0.043	5.511	0.000	0.153	0.323
Target type: Self	-1.457	0.080	-18.271	0.000	-1.614	-1.301
Target type: Young	-1.066	0.066	-16.231	0.000	-1.195	-0.937
Graph Literacy (Centered)	-0.148	0.058	-2.550	0.011	-0.261	-0.034
COVID-19 Knowledge (Centered)	-0.085	0.045	-1.885	0.059	-0.174	0.003
Health Risk Factors (Centered)	0.161	0.068	2.370	0.018	0.028	0.293
Contracted COVID-19 (Centered)	0.369	0.089	4.147	0.000	0.195	0.543
Tested for COVID-19 (Centered)	-0.075	0.075	-1.008	0.313	-0.222	0.071
Age (Centered)	-0.011	0.006	-1.969	0.049	-0.022	0.000
Gender (Centered)	0.011	0.064	0.167	0.867	-0.114	0.136
Education (Centered)	0.058	0.039	1.488	0.137	-0.018	0.135
Time point *Y-axis	-0.374	0.078	-4.794	0.000	-0.527	-0.221

4.1.5.3 Model with only participants in the 6 Models group

This model had a significant effect of time point highlighted in green below.

	Estimate	Std..Error	t.value	p.z	2.5 %	97.5 %
(Intercept)	4.954	0.111	44.493	0.000	4.736	5.173
**Time point**	**0.237**	**0.053**	**4.502**	**0.000**	**0.134**	**0.341**
Y-axis	-0.053	0.130	-0.408	0.684	-0.308	0.202
Question type	0.114	0.044	2.624	0.009	0.029	0.200
Target type: Self	-1.361	0.086	-15.760	0.000	-1.531	-1.192
Target type: Young	-1.080	0.067	-16.096	0.000	-1.212	-0.949
Graph Literacy (Centered)	-0.224	0.060	-3.737	0.000	-0.341	-0.106
COVID-19 Knowledge (Centered)	0.061	0.034	1.785	0.074	-0.006	0.129
Health Risk Factors (Centered)	0.104	0.055	1.879	0.060	-0.004	0.212
Contracted COVID-19 (Centered)	0.170	0.088	1.945	0.052	-0.001	0.342
Tested for COVID-19 (Centered)	0.245	0.070	3.518	0.000	0.108	0.381
Age (Centered)	0.000	0.005	0.046	0.963	-0.010	0.011
Gender (Centered)	-0.070	0.062	-1.126	0.260	-0.191	0.052
Education (Centered)	0.007	0.041	0.169	0.866	-0.074	0.088
Time point *Y-axis	-0.232	0.074	-3.129	0.002	-0.377	-0.087

4.1.5.4 Model with only participants in the CI95 group

This model had a significant effect of time point highlighted in green below.

	Estimate	Std..Error	t.value	p.z	2.5 %	97.5 %
(Intercept)	4.958	0.130	38.147	0.000	4.703	5.213
**Time point**	**0.254**	**0.048**	**5.326**	**0.000**	**0.161**	**0.348**
Y-axis	-0.352	0.160	-2.200	0.028	-0.666	-0.038
Question type	0.192	0.038	5.104	0.000	0.119	0.266
Target type: Self	-1.223	0.085	-14.368	0.000	-1.390	-1.056
Target type: Young	-0.938	0.068	-13.788	0.000	-1.071	-0.805
Graph Literacy (Centered)	-0.080	0.073	-1.100	0.271	-0.223	0.063
COVID-19 Knowledge (Centered)	-0.052	0.035	-1.480	0.139	-0.122	0.017
Health Risk Factors (Centered)	0.219	0.066	3.300	0.001	0.089	0.349
Contracted COVID-19 (Centered)	0.191	0.104	1.835	0.066	-0.013	0.395
Tested for COVID-19 (Centered)	0.122	0.084	1.449	0.147	-0.043	0.286
Age (Centered)	-0.005	0.007	-0.749	0.454	-0.018	0.008
Gender (Centered)	-0.050	0.076	-0.653	0.514	-0.198	0.099
Education (Centered)	0.042	0.046	0.908	0.364	-0.049	0.133
Time point *Y-axis	-0.332	0.068	-4.900	0.000	-0.465	-0.199

4.1.5.5 Model with only participants in the 3 Models CI95 group

This model had a significant effect of time point highlighted in green below.

	Estimate	Std..Error	t.value	p.z	2.5 %	97.5 %
(Intercept)	4.792	0.129	37.241	0.000	4.540	5.044
**Time point**	**0.202**	**0.050**	**4.058**	**0.000**	**0.105**	**0.300**
Y-axis	-0.069	0.149	-0.462	0.644	-0.361	0.224
Question type	0.169	0.043	3.960	0.000	0.085	0.253
Target type: Self	-1.310	0.080	-16.305	0.000	-1.468	-1.153
Target type: Young	-0.867	0.064	-13.642	0.000	-0.991	-0.742
Graph Literacy (Centered)	-0.167	0.068	-2.459	0.014	-0.300	-0.034
COVID-19 Knowledge (Centered)	-0.127	0.039	-3.279	0.001	-0.203	-0.051
Health Risk Factors (Centered)	-0.013	0.039	-0.341	0.733	-0.089	0.062
Contracted COVID-19 (Centered)	-0.059	0.107	-0.555	0.579	-0.269	0.150
Tested for COVID-19 (Centered)	0.050	0.081	0.610	0.542	-0.110	0.209
Age (Centered)	0.006	0.007	0.880	0.379	-0.007	0.019
Gender (Centered)	0.035	0.073	0.475	0.635	-0.108	0.177
Education (Centered)	0.008	0.043	0.186	0.853	-0.076	0.092
Time point *Y-axis	-0.243	0.071	-3.433	0.001	-0.381	-0.104

4.1.6 Post hoc test: Negative effect of time point for CI 50 incident data

Finally, we noticed that CI 50 produced a negative change in risk estimates with an incident data (see Figure 4.1.2 bottom). To determine if the CI 50 produced a reliably negative change in risk estimates compared to the other visualizations, we ran a post hoc analysis with just the incident data and CI 50 as the referent (Table below). The model provided evidence for a negative impact of time point for CI 50 (b = -.22, SD = .06, p = .000, CIs[-.34, -.11]) (highlighted in green) and interactions with 6 Models (b = .24, SD = .09 , p = .006, CIs[.07, .40]) and 3 Models showing the range of CI 95 (b = .20, SD = .09, p = .019, CIs[.03, .37]) (highlighted in blue). We ran follow-up analyses with the 6 Models (Table 4.1.6.1) and 3 Models CI 95 (Table 4.1.6.2) as the referents, which showed no evidence for an effect of time point (highlighted in green). This analysis suggested that participants who viewed incident COVID-19 death data with a forecast showing a mean with 50% confidence intervals significantly decreased their risk ratings compared to participants who viewed forecasts with 6 Models or 3 Models showing a 95% confidence interval range.

	Estimate	Std..Error	t.value	p.z	2.5 %	97.5 %
(Intercept)	4.766	0.102	46.553	0.000	4.565	4.967
**Time point**	**-0.224**	**0.061**	**-3.681**	**0.000**	**-0.343**	**-0.105**
Visualization: No Forecast	-0.165	0.133	-1.247	0.213	-0.425	0.095
Visualization: All Models	0.017	0.134	0.128	0.898	-0.245	0.279
Visualization: 6 Models	0.018	0.132	0.137	0.891	-0.241	0.277
Visualization: 3 Models CI 95	0.156	0.132	1.182	0.237	-0.103	0.415
Visualization: 3 Models CI 50	-0.018	0.132	-0.137	0.891	-0.277	0.241
Visualization: CI 95	-0.227	0.132	-1.727	0.084	-0.485	0.031
Visualization: Mean	0.080	0.131	0.610	0.542	-0.177	0.338
Question type	0.229	0.021	10.869	0.000	0.188	0.270
Target type: Self	-1.412	0.039	-36.573	0.000	-1.487	-1.336
Target type: Young	-1.035	0.032	-31.931	0.000	-1.099	-0.972
Graph Literacy (Centered)	-0.153	0.030	-5.164	0.000	-0.211	-0.095
COVID-19 Knowledge (Centered)	-0.038	0.018	-2.138	0.032	-0.073	-0.003
Contracted COVID-19 (Centered)	0.117	0.051	2.291	0.022	0.017	0.217
Tested for COVID-19 (Centered)	0.020	0.036	0.539	0.590	-0.051	0.090
Health Risk Factors (Centered)	0.112	0.030	3.675	0.000	0.052	0.172
Age (Centered)	-0.002	0.003	-0.673	0.501	-0.008	0.004
Gender (Centered)	0.043	0.033	1.285	0.199	-0.022	0.108
Education (Centered)	0.007	0.020	0.326	0.745	-0.033	0.047
Time point *Visualization: No Forecast	0.163	0.087	1.876	0.061	-0.007	0.332
Time point *Visualization: All Models	0.148	0.087	1.709	0.087	-0.022	0.318
**Time point *Visualization: 6 Models**	**0.235**	**0.086**	**2.734**	**0.006**	**0.067**	**0.404**
**Time point *Visualization: 3 Models CI 95**	**0.202**	**0.086**	**2.343**	**0.019**	**0.033**	**0.370**
Time point *Visualization: 3 Models CI 50	0.090	0.086	1.048	0.295	-0.078	0.259
Time point *Visualization: CI 95	0.143	0.086	1.663	0.096	-0.026	0.312
Time point *Visualization: Mean	0.110	0.086	1.284	0.199	-0.058	0.279

4.1.6.1 Model with 6 Models as the referent and incident data This model revealed no evidence for an effect of time point (highlighted in green).

	Estimate	Std..Error	t.value	p.z	2.5 %	97.5 %
(Intercept)	4.784	0.100	47.768	0.000	4.588	4.980
**Time point**	**0.012**	**0.061**	**0.190**	**0.849**	**-0.108**	**0.132**
Visualization: CI 50	-0.018	0.132	-0.137	0.891	-0.277	0.241
Visualization: No Forecast	-0.183	0.133	-1.381	0.167	-0.444	0.077
Visualization: All Models	-0.001	0.133	-0.008	0.994	-0.261	0.259
Visualization: 3 Models CI 95	0.138	0.132	1.042	0.297	-0.121	0.397
Visualization: 3 Models CI 50	-0.036	0.132	-0.275	0.783	-0.294	0.222
Visualization: CI 95	-0.245	0.132	-1.856	0.063	-0.505	0.014
Visualization: Mean	0.062	0.133	0.468	0.640	-0.198	0.322
Question type	0.229	0.021	10.869	0.000	0.188	0.270
Target type: Self	-1.412	0.039	-36.574	0.000	-1.487	-1.336
Target type: Young	-1.035	0.032	-31.932	0.000	-1.099	-0.972
Graph Literacy (Centered)	-0.153	0.030	-5.164	0.000	-0.211	-0.095
COVID-19 Knowledge (Centered)	-0.038	0.018	-2.138	0.032	-0.073	-0.003
Contracted COVID-19 (Centered)	0.117	0.051	2.291	0.022	0.017	0.217
Tested for COVID-19 (Centered)	0.020	0.036	0.539	0.590	-0.051	0.090
Health Risk Factors (Centered)	0.112	0.030	3.675	0.000	0.052	0.172
Age (Centered)	-0.002	0.003	-0.673	0.501	-0.008	0.004
Gender (Centered)	0.043	0.033	1.285	0.199	-0.022	0.108
Education (Centered)	0.007	0.020	0.326	0.745	-0.033	0.047
Time point *Visualization: CI 50	-0.235	0.086	-2.734	0.006	-0.404	-0.067
Time point *Visualization: No Forecast	-0.073	0.087	-0.836	0.403	-0.243	0.098
Time point *Visualization: All Models	-0.087	0.087	-1.003	0.316	-0.258	0.083
Time point *Visualization: 3 Models CI 95	-0.034	0.086	-0.389	0.697	-0.203	0.136
Time point *Visualization: 3 Models CI 50	-0.145	0.086	-1.680	0.093	-0.314	0.024
Time point *Visualization: CI 95	-0.092	0.086	-1.067	0.286	-0.261	0.077
Time point *Visualization: Mean	-0.125	0.086	-1.445	0.148	-0.294	0.044

4.1.6.2 Model with 3 Model CI95 as the referent and incident data This model revealed no evidence for an effect of time point (highlighted in green).

	Estimate	Std..Error	t.value	p.z	2.5 %	97.5 %
(Intercept)	4.922	0.102	48.443	0.000	4.723	5.121
**Time point**	**-0.022**	**0.061**	**-0.359**	**0.719**	**-0.142**	**0.098**
Visualization: 6 Models	-0.138	0.132	-1.042	0.297	-0.397	0.121
Visualization: CI 50	-0.156	0.132	-1.182	0.237	-0.415	0.103
Visualization: No Forecast	-0.321	0.133	-2.415	0.016	-0.582	-0.061
Visualization: All Models	-0.139	0.134	-1.039	0.299	-0.401	0.123
Visualization: 3 Models CI 50	-0.174	0.132	-1.316	0.188	-0.434	0.085
Visualization: CI 95	-0.383	0.132	-2.904	0.004	-0.642	-0.125
Visualization: Mean	-0.076	0.132	-0.574	0.566	-0.335	0.183
Question type	0.229	0.021	10.869	0.000	0.188	0.270
Target type: Self	-1.412	0.039	-36.575	0.000	-1.487	-1.336
Target type: Young	-1.035	0.032	-31.933	0.000	-1.099	-0.972
Graph Literacy (Centered)	-0.153	0.030	-5.163	0.000	-0.211	-0.095
COVID-19 Knowledge (Centered)	-0.038	0.018	-2.138	0.032	-0.073	-0.003
Contracted COVID-19 (Centered)	0.117	0.051	2.291	0.022	0.017	0.217
Tested for COVID-19 (Centered)	0.020	0.036	0.539	0.590	-0.051	0.090
Health Risk Factors (Centered)	0.112	0.030	3.675	0.000	0.052	0.172
Age (Centered)	-0.002	0.003	-0.673	0.501	-0.008	0.004
Gender (Centered)	0.043	0.033	1.285	0.199	-0.022	0.108
Education (Centered)	0.007	0.020	0.326	0.745	-0.033	0.047
Time point *Visualization: 6 Models	0.034	0.086	0.389	0.697	-0.136	0.203
Time point *Visualization: CI 50	-0.202	0.086	-2.343	0.019	-0.370	-0.033
Time point *Visualization: No Forecast	-0.039	0.087	-0.450	0.653	-0.210	0.131
Time point *Visualization: All Models	-0.054	0.087	-0.616	0.538	-0.224	0.117
Time point *Visualization: 3 Models CI 50	-0.111	0.086	-1.291	0.197	-0.281	0.058
Time point *Visualization: CI 95	-0.059	0.086	-0.678	0.498	-0.228	0.111
Time point *Visualization: Mean	-0.091	0.086	-1.056	0.291	-0.260	0.078

4.2 Exp 2

We ran a second experiment in December of 2020 with participants in California and New York. The analysis of the second experiment was conducted in two steps. First, we tested a subset of the visualizations that produced the more notable findings in Experiment 1, including No Forecast (as a control), CI 50, and 6 Models.

For the second step, we tested three new visualization techniques; a gradient visualizations with and without a mean line and CI 95 without a centerline. We tested the three new visualizations with only participants in California.

4.2.1 Model 1 (CA vs. NY)

The first analysis used the previously describe multi-level modeling procedure that included the California incident, California cumulative, New York incident, and New York cumulative y-axes as a four-level predictor termed *StateData* in the model (see model description below).

Risk estimate (outcome) ∼
Time point * Visualization (fixed-interaction) +
Time point * StateData (fixed-interaction) +
State Data * Visualization (fixed-interaction) +
Time point (fixed-effect, categorical 2 levels, referent = pre-visualization exposure) +
Visualization (fixed-effect, categorical 3 levels, referent = No Forecast) +
State Data (fixed-effect, categorical 4 levels, referent = CA incident) +
Question type (fixed-effect, numeric, coded as -1, -.5, .5, 1) +
Target type (fixed-effect, categorical 3 levels, referent = self) +
Graph literacy centered centered (fixed-effect, numeric 0-4, centered on grand mean) +
COVID-19-knowlege centered (fixed-effect, numeric 1-13, centered on grand mean) +
COVID-19-health-risk centered (fixed-effect, numeric 1-20, centered on grand mean) +
Contracted COVID-19 centered (fixed-effect, dichotomous, yes = 1 no = -1) +
Tested for COVID-19 centered (fixed-effect, dichotomous, yes = 1 no = -1) +
Age centered (fixed-effect, numeric, centered on grand mean)+
Gender centered (fixed-effect, dichotomous, male = 1 female = -1) +
Education centered (fixed-effect, numeric 1-8, centered on grand mean) +
(Target type + Question type + Time point | Participant Id)

This analyses revealed three interactions (see Table below, highlighted in blue). The first two were between time point * California incident data vs. New York incident data (b = -0.24, *SD* = .05, p = .000, *CIs*[-0.34, -0.14]) and time point * California incident data vs. New York cumulative (b = -0.16, *SD* = .051, p = .002, *CIs*[-0.26, -0.06]), means shown in the dashed lines of Figure 4.2.2.

The third interaction revealed by the omnibus analysis was between time point and No Forecast vs. 6 Models (b = .12, *SD* = .04, p = .008, *CIs*[.03, .20]). We will break down these interactions in Section 4.2.3.1.

	Estimate	Std..Error	t.value	p.z	2.5 %	97.5 %
(Intercept)	4.819	0.096	50.329	0.000	4.631	5.006
State Data: CA cumulative	-0.126	0.126	-1.000	0.317	-0.372	0.121
State Data: NY incident	-0.263	0.126	-2.082	0.037	-0.511	-0.015
State Data: NY cumulative	-0.053	0.125	-0.423	0.673	-0.299	0.193
Time point	0.246	0.045	5.498	0.000	0.158	0.334
Visualization: CI 50	0.164	0.126	1.299	0.194	-0.083	0.410
Visualization: 6 Models	0.077	0.125	0.610	0.542	-0.169	0.322
Question type	0.139	0.019	7.509	0.000	0.103	0.176
Target type: Self	-1.583	0.032	-48.786	0.000	-1.647	-1.520
Target type: Young	-1.072	0.028	-38.676	0.000	-1.127	-1.018
Graph Literacy (Centered)	-0.128	0.024	-5.289	0.000	-0.175	-0.081
COVID-19 Knowledge (Centered)	-0.025	0.016	-1.534	0.125	-0.057	0.007
Health Risk Factors (Centered)	0.106	0.031	3.426	0.001	0.045	0.167
Contracted COVID-19 (Centered)	0.034	0.037	0.934	0.350	-0.038	0.106
Tested for COVID-19 (Centered)	0.071	0.026	2.719	0.007	0.020	0.122
Age (Centered)	-0.004	0.003	-1.635	0.102	-0.009	0.001
Gender (Centered)	-0.070	0.026	-2.718	0.007	-0.120	-0.019
Education (Centered)	0.027	0.016	1.710	0.087	-0.004	0.058
State Data: CA cumulative *Time point	-0.027	0.051	-0.529	0.597	-0.128	0.073
**State Data: NY incident *Time point**	**-0.241**	**0.051**	**-4.692**	**0.000**	**-0.341**	**-0.140**
**State Data: NY cumulative *Time point**	**-0.163**	**0.051**	**-3.166**	**0.002**	**-0.264**	**-0.062**
Time point *Visualization: CI 50	0.058	0.044	1.309	0.191	-0.029	0.145
**Time point *Visualization: 6 Models**	**0.117**	**0.044**	**2.639**	**0.008**	**0.030**	**0.204**
State Data: CA cumulative *Visualization: CI 50	-0.033	0.177	-0.188	0.851	-0.379	0.313
State Data: NY incident *Visualization: CI 50	0.026	0.177	0.146	0.884	-0.321	0.372
State Data: NY cumulative *Visualization: CI 50	-0.241	0.176	-1.364	0.172	-0.586	0.105
State Data: CA cumulative *Visualization: 6 Models	-0.051	0.176	-0.292	0.771	-0.396	0.293
State Data: NY incident *Visualization: 6 Models	0.044	0.176	0.248	0.804	-0.302	0.390
State Data: NY cumulative *Visualization: 6 Models	-0.141	0.176	-0.801	0.423	-0.486	0.204

Effect size for the model:

```
##             R2m       R2c
## [1,] 0.1945742 0.5056568
```

4.2.1.1 Mean risk judgments for each condition

Table showing summary statics for each condition

Visualization	StateData	Time-point	N	Mean Judgment	sd	se	ci
6 Models	CA cumulative	Pre	900	3.774	2.105	0.070	0.138
6 Models	CA cumulative	Post	900	4.154	2.004	0.067	0.131
6 Models	CA incident	Pre	900	3.941	1.974	0.066	0.129
6 Models	CA incident	Post	900	4.399	1.821	0.061	0.119
6 Models	NY cumulative	Pre	900	3.734	2.158	0.072	0.141
6 Models	NY cumulative	Post	900	3.874	2.055	0.068	0.134
6 Models	NY incident	Pre	900	3.810	2.028	0.068	0.133
6 Models	NY incident	Post	900	3.869	1.916	0.064	0.125
CI 50	CA cumulative	Pre	900	3.870	1.992	0.066	0.130
CI 50	CA cumulative	Post	900	4.143	1.871	0.062	0.122
CI 50	CA incident	Pre	900	4.037	2.142	0.071	0.140
CI 50	CA incident	Post	900	4.291	2.055	0.069	0.134
CI 50	NY cumulative	Pre	900	3.818	1.987	0.066	0.130
CI 50	NY cumulative	Post	900	3.939	1.913	0.064	0.125
CI 50	NY incident	Pre	900	3.807	2.030	0.068	0.133
CI 50	NY incident	Post	900	3.958	1.873	0.062	0.122
No Forecast	CA cumulative	Pre	900	3.753	2.124	0.071	0.139
No Forecast	CA cumulative	Post	900	3.946	2.027	0.068	0.133
No Forecast	CA incident	Pre	900	3.909	2.114	0.070	0.138
No Forecast	CA incident	Post	900	4.138	2.023	0.067	0.132
No Forecast	NY cumulative	Pre	900	3.828	2.035	0.068	0.133
No Forecast	NY cumulative	Post	900	3.987	1.928	0.064	0.126
No Forecast	NY incident	Pre	900	3.609	2.014	0.067	0.132
No Forecast	NY incident	Post	900	3.597	1.839	0.061	0.120

4.2.2 Plot of primary task results

Results of the trend comparison in Experiment 2, where pre-visualization risk judgments are colored gray and post-visualization judgments blue, for cumulative y-axis (top) and incident y-axis (bottom). Dashed lines show the mean pre-and post-visualization risk judgments for each y-axis group as a whole. Black bars show 95% confidence intervals around the mean (black dot) for each condition using the Cousineau-Morey method and the density plots were generated from this data.

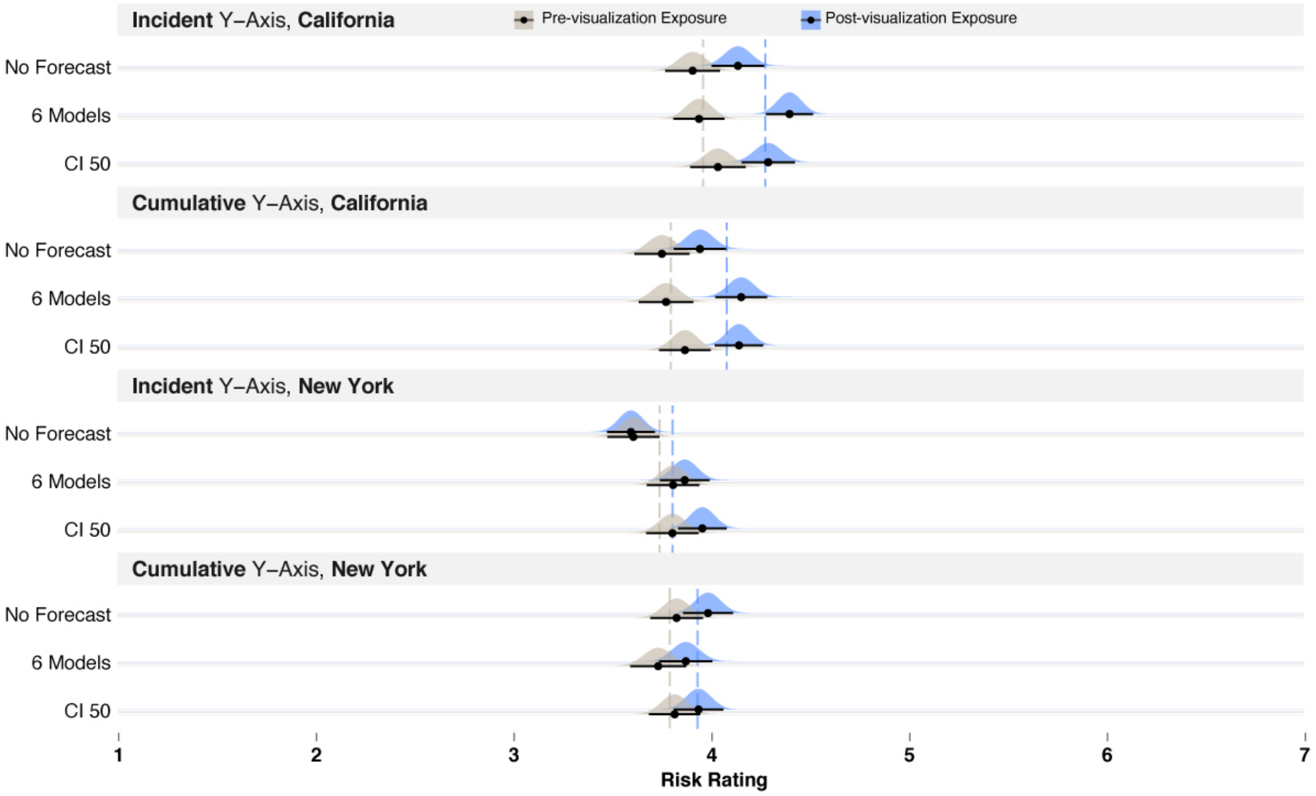

4.2.3 Interaction analysis: Time point * CA cumulative, NY incident, NY cumulative

To break down these interactions we ran separate models for California incident (Table 4.2.3.1), New York cumulative (Table 4.2.3.2), and New York incident (Table 4.2.3.3).

4.2.3.1 Model using California incident data and No Forecast as the referent These analysis revealed a large effect of time point for California incident data ($b = .22$, $SD = .06$, $p = .000$, $CIs[.1, .33]$; highlighted in green).

	Estimate	Std..Error	t.value	p.z	2.5 %	97.5 %
(Intercept)	4.832	0.121	39.796	0.000	4.594	5.070
**Time point**	**0.216**	**0.060**	**3.593**	**0.000**	**0.098**	**0.333**
Visualization: CI 50	0.166	0.135	1.235	0.217	-0.098	0.431
Visualization: 6 Models	0.038	0.134	0.280	0.780	-0.225	0.300
Question type	0.113	0.039	2.918	0.004	0.037	0.189
Target type: Self	-1.618	0.067	-24.029	0.000	-1.750	-1.486
Target type: Young	-1.048	0.056	-18.845	0.000	-1.157	-0.939
Graph Literacy (Centered)	-0.181	0.053	-3.450	0.001	-0.284	-0.078
COVID-19 Knowledge (Centered)	0.012	0.032	0.373	0.709	-0.051	0.075
Health Risk Factors (Centered)	-0.012	0.066	-0.175	0.861	-0.142	0.119
Contracted COVID-19 (Centered)	0.060	0.086	0.698	0.485	-0.109	0.229
Tested for COVID-19 (Centered)	0.071	0.058	1.220	0.222	-0.043	0.184
Age (Centered)	-0.002	0.005	-0.318	0.751	-0.012	0.008
Gender (Centered)	-0.047	0.056	-0.845	0.398	-0.156	0.062
Education (Centered)	0.007	0.034	0.200	0.841	-0.060	0.074
Time point *Visualization: CI 50	0.025	0.084	0.294	0.768	-0.140	0.189
Time point *Visualization: 6 Models	0.241	0.084	2.881	0.004	0.077	0.405

4.2.3.2 Model using New York cumulative data and No Forecast as the referent There was less of an effect of time point for New York cumulative data ($b = .17$, $SD = .06$, $p = .009$, $CIs[.04, .29]$; highlighted in green) than California incident.

	Estimate	Std..Error	t.value	p.z	2.5 %	97.5 %
(Intercept)	4.680	0.105	44.501	0.000	4.474	4.886
**Time point**	**0.166**	**0.063**	**2.628**	**0.009**	**0.042**	**0.290**
Visualization: CI 50	-0.007	0.124	-0.060	0.952	-0.250	0.235
Visualization: 6 Models	0.028	0.123	0.231	0.818	-0.213	0.269
Question type	0.107	0.034	3.196	0.001	0.041	0.173
Target type: Self	-1.667	0.065	-25.476	0.000	-1.795	-1.539
Target type: Young	-1.153	0.057	-20.186	0.000	-1.265	-1.041
Graph Literacy (Centered)	-0.087	0.047	-1.842	0.066	-0.179	0.006
COVID-19 Knowledge (Centered)	-0.060	0.030	-1.960	0.050	-0.120	0.000
Health Risk Factors (Centered)	0.171	0.065	2.658	0.008	0.045	0.298
Contracted COVID-19 (Centered)	-0.098	0.069	-1.406	0.160	-0.234	0.038
Tested for COVID-19 (Centered)	0.063	0.051	1.243	0.214	-0.036	0.163
Age (Centered)	0.005	0.005	1.037	0.300	-0.005	0.015
Gender (Centered)	0.040	0.050	0.800	0.424	-0.058	0.139
Education (Centered)	0.034	0.031	1.085	0.278	-0.027	0.095
Time point *Visualization: CI 50	-0.033	0.089	-0.365	0.715	-0.208	0.143
Time point *Visualization: 6 Models	-0.051	0.090	-0.565	0.572	-0.226	0.125

4.2.3.3 Model using New York incident data and No Forecast as the referent

There was no evidence for an effect of time point for New York incident data (b = -0.02, SD = .06, p = .79, CIs[-.13, .1]; highlighted in green).

	Estimate	Std..Error	t.value	p.z	2.5 %	97.5 %
(Intercept)	4.527	0.100	45.239	0.000	4.330	4.723
**Time point**	**-0.016**	**0.059**	**-0.262**	**0.793**	**-0.132**	**0.101**
Visualization: CI 50	0.183	0.119	1.543	0.123	-0.049	0.416
Visualization: 6 Models	0.128	0.118	1.088	0.277	-0.103	0.360
Question type	0.187	0.037	5.052	0.000	0.115	0.260
Target type: Self	-1.538	0.062	-24.824	0.000	-1.659	-1.416
Target type: Young	-1.100	0.052	-21.192	0.000	-1.202	-0.998
Graph Literacy (Centered)	-0.093	0.044	-2.126	0.034	-0.179	-0.007
COVID-19 Knowledge (Centered)	-0.034	0.033	-1.016	0.309	-0.099	0.031
Health Risk Factors (Centered)	0.197	0.052	3.811	0.000	0.095	0.298
Contracted COVID-19 (Centered)	-0.022	0.068	-0.329	0.742	-0.156	0.111
Tested for COVID-19 (Centered)	0.065	0.049	1.333	0.182	-0.031	0.161
Age (Centered)	-0.012	0.004	-2.696	0.007	-0.021	-0.003
Gender (Centered)	-0.084	0.048	-1.731	0.083	-0.179	0.011
Education (Centered)	0.033	0.029	1.148	0.251	-0.023	0.089
Time point *Visualization: CI 50	0.167	0.083	2.002	0.045	0.004	0.330
Time point *Visualization: 6 Models	0.070	0.083	0.848	0.396	-0.092	0.233

To determine if New York cumulative data produced greater risk estimates compared to New York incident data we ran the prior omnibus model with New York cumulative data as the referent (Table below). This analysis did not reveal evidence for an interaction between time point and New York cumulative vs. incident data (b = -0.08, SD = .05, p = .13, CIs[-.18, .02], highlighted in blue), meaning that we did not find evidence that New York cumulative and incident data evoke differential risk estimates.

	Estimate	Std..Error	t.value	p.z	2.5 %	97.5 %
(Intercept)	4.766	0.093	51.464	0.000	4.584	4.947
State Data: CA incident	0.053	0.125	0.423	0.673	-0.193	0.299
State Data: CA cumulative	-0.073	0.125	-0.584	0.559	-0.317	0.172
State Data: NY incident	-0.210	0.125	-1.685	0.092	-0.455	0.034
Time point	0.083	0.044	1.878	0.060	-0.004	0.170
Visualization: CI 50	-0.077	0.124	-0.619	0.536	-0.321	0.167
Visualization: 6 Models	-0.065	0.125	-0.518	0.604	-0.309	0.180
Question type	0.139	0.019	7.509	0.000	0.103	0.176
Target type: Self	-1.583	0.032	-48.786	0.000	-1.647	-1.520
Target type: Young	-1.072	0.028	-38.676	0.000	-1.127	-1.018
Graph Literacy (Centered)	-0.128	0.024	-5.289	0.000	-0.175	-0.081
COVID-19 Knowledge (Centered)	-0.025	0.016	-1.534	0.125	-0.057	0.007
Health Risk Factors (Centered)	0.106	0.031	3.426	0.001	0.045	0.167
Contracted COVID-19 (Centered)	0.034	0.037	0.934	0.350	-0.038	0.106
Tested for COVID-19 (Centered)	0.071	0.026	2.719	0.007	0.020	0.122
Age (Centered)	-0.004	0.003	-1.635	0.102	-0.009	0.001
Gender (Centered)	-0.070	0.026	-2.718	0.007	-0.120	-0.019
Education (Centered)	0.027	0.016	1.710	0.087	-0.004	0.058
State Data: CA incident *Time point	0.163	0.051	3.166	0.002	0.062	0.264
State Data: CA cumulative *Time point	0.136	0.051	2.654	0.008	0.036	0.236
**State Data: NY incident *Time point**	**-0.078**	**0.051**	**-1.522**	**0.128**	**-0.178**	**0.022**
Time point *Visualization: CI 50	0.058	0.044	1.309	0.191	-0.029	0.145
Time point *Visualization: 6 Models	0.117	0.044	2.639	0.008	0.030	0.204
State Data: CA incident *Visualization: CI 50	0.241	0.176	1.364	0.173	-0.105	0.586
State Data: CA cumulative *Visualization: CI 50	0.207	0.176	1.181	0.238	-0.137	0.552
State Data: NY incident *Visualization: CI 50	0.266	0.176	1.516	0.130	-0.078	0.611
State Data: CA incident *Visualization: 6 Models	0.141	0.176	0.801	0.423	-0.204	0.486
State Data: CA cumulative *Visualization: 6 Models	0.090	0.175	0.512	0.609	-0.254	0.434
State Data: NY incident *Visualization: 6 Models	0.185	0.176	1.051	0.293	-0.160	0.530

4.2.4 Interaction analysis: Time Point * No Forecast vs. 6 Models

The third interaction revealed by the omnibus analysis was between time point and No Forecast vs. 6 Models (b = .12, SD = .04, p = .008, CIs[.03, .20]). To break down this interaction we ran separate models for 6 Models (Table 4.2.4.1) (pre-visualization exposure m = 3.815, SD = 2.069; post-visualization exposure m = 4.07, SD = 1.95, change = .26) and No Forecast (Table 4.2.4.2) (pre-visualization exposure m = 3.77, SD = 2.07; post-visualization exposure m = 3.91, SD = 1.96, change = .14).

4.2.4.1 Model with participants in the 6 Models group This analysis provided evidence that 6 Models had a larger effect of time point (b = .47, SD = .07, p = .000, CIs[.34, .60], Table below highlighted in green) compared to No Forecast.

	Estimate	Std..Error	t.value	p.z	2.5 %	97.5 %
(Intercept)	4.847	0.098	49.585	0.000	4.656	5.039
**Time point**	**0.468**	**0.066**	**7.139**	**0.000**	**0.340**	**0.597**
State Data: CA cumulative	-0.192	0.118	-1.631	0.103	-0.423	0.039
State Data: NY incident	-0.207	0.120	-1.720	0.085	-0.442	0.029
State Data: NY cumulative	-0.148	0.120	-1.239	0.216	-0.382	0.086
Question type	0.164	0.033	4.893	0.000	0.098	0.229
Target type: Self	-1.571	0.060	-26.088	0.000	-1.689	-1.453
Target type: Young	-1.023	0.048	-21.469	0.000	-1.117	-0.930
Graph Literacy (Centered)	-0.087	0.041	-2.143	0.032	-0.167	-0.007
COVID-19 Knowledge (Centered)	-0.058	0.027	-2.159	0.031	-0.112	-0.005
Health Risk Factors (Centered)	0.151	0.048	3.112	0.002	0.056	0.246
Contracted COVID-19 (Centered)	0.006	0.060	0.104	0.917	-0.111	0.123
Tested for COVID-19 (Centered)	0.064	0.043	1.483	0.138	-0.021	0.149
Age (Centered)	-0.006	0.004	-1.364	0.173	-0.014	0.003
Gender (Centered)	-0.121	0.043	-2.831	0.005	-0.205	-0.037
Education (Centered)	0.055	0.026	2.076	0.038	0.003	0.106
Time point *State Data: CA cumulative	-0.087	0.092	-0.946	0.344	-0.269	0.094
Time point *State Data: NY incident	-0.407	0.092	-4.401	0.000	-0.588	-0.226
Time point *State Data: NY cumulative	-0.353	0.093	-3.776	0.000	-0.535	-0.170

4.2.4.2 Model with participants in the No Forecast group The model with participants in the No Forecast groups showed a smaller effect of time point (b = .22, SD = .07, p = .001, CIs[.09, .35], Table below highlighted in green).

	Estimate	Std..Error	t.value	p.z	2.5 %	97.5 %
(Intercept)	4.860	0.108	44.801	0.000	4.647	5.072
**Time point**	**0.220**	**0.066**	**3.331**	**0.001**	**0.091**	**0.350**
State Data: CA cumulative	-0.151	0.128	-1.182	0.237	-0.401	0.099
State Data: NY incident	-0.270	0.129	-2.100	0.036	-0.522	-0.018
State Data: NY cumulative	-0.088	0.127	-0.691	0.490	-0.337	0.161
Question type	0.126	0.031	4.040	0.000	0.065	0.187
Target type: Self	-1.538	0.054	-28.500	0.000	-1.644	-1.433
Target type: Young	-1.086	0.047	-22.920	0.000	-1.179	-0.993
Graph Literacy (Centered)	-0.090	0.040	-2.258	0.024	-0.168	-0.012
COVID-19 Knowledge (Centered)	-0.025	0.027	-0.902	0.367	-0.078	0.029
Health Risk Factors (Centered)	0.129	0.058	2.231	0.026	0.016	0.242
Contracted COVID-19 (Centered)	0.078	0.065	1.198	0.231	-0.050	0.206
Tested for COVID-19 (Centered)	0.085	0.046	1.850	0.064	-0.005	0.176
Age (Centered)	-0.002	0.005	-0.377	0.706	-0.011	0.007
Gender (Centered)	-0.003	0.046	-0.070	0.944	-0.093	0.086
Education (Centered)	-0.023	0.028	-0.792	0.428	-0.078	0.033
Time point *State Data: CA cumulative	-0.033	0.092	-0.359	0.719	-0.214	0.148
Time point *State Data: NY incident	-0.246	0.093	-2.648	0.008	-0.427	-0.064
Time point *State Data: NY cumulative	-0.055	0.092	-0.598	0.550	-0.236	0.125

4.2.5 Model 2 (CA additional visualizations)

The goal of the second analysis was to examine the impact of the three new visualization techniques which were shown to participants in California. We specified 6 Models as the referent because it showed the largest increase in risk ratings in the last analysis. This analysis revealed interactions between time point and 6 Models vs. all of the other visualizations (see Table below highlighted in blue).

Risk estimate (outcome) ∼
Time point * Visualization (fixed-interaction) +
Time point * Y-axis (fixed-interaction) +
Y-axis * Visualization (fixed-interaction) +
Time point (fixed-effect, categorical 2 levels, referent = pre-visualization exposure) +
Visualization (fixed-effect, categorical 6 levels, referent = 6-model) +
Y-axis (fixed-effect, categorical 2 levels, referent = cumulative) +
Question type (fixed-effect, numeric, coded as -1, -.5, .5, 1) +
Target type (fixed-effect, categorical 3 levels, referent = self) +
Graph literacy centered centered (fixed-effect, numeric 0-4, centered on grand mean) +
COVID-19-knowlege centered (fixed-effect, numeric 1-13, centered on grand mean) +
COVID-19-health-risk centered (fixed-effect, numeric 1-20, centered on grand mean) +
Contracted COVID-19 centered (fixed-effect, dichotomous, yes = 1 no = -1) +
Tested for COVID-19 centered (fixed-effect, dichotomous, yes = 1 no = -1) +
Age centered (fixed-effect, numeric, centered on grand mean)+
Gender centered (fixed-effect, dichotomous, male = 1 female = -1) +
Education centered (fixed-effect, numeric 1-8, centered on grand mean) +
(Target type + Question type + Time point | Participant Id)

	Estimate	Std..Error	t.value	p.z	2.5 %	97.5 %
(Intercept)	4.674	0.096	48.906	0.000	4.487	4.861
Time point	0.428	0.046	9.405	0.000	0.339	0.518
Visualization: No Forecast	-0.028	0.127	-0.216	0.829	-0.277	0.222
Visualization: CI 50	0.119	0.127	0.938	0.348	-0.130	0.369
Visualization: Gradient	0.189	0.127	1.490	0.136	-0.060	0.438
Visualization: Gradient+Mean	0.048	0.127	0.374	0.709	-0.202	0.297
Visualization: CI95 No Mean	-0.081	0.127	-0.641	0.522	-0.329	0.167
Y-axis	0.180	0.127	1.422	0.155	-0.068	0.429
Question type	0.123	0.019	6.498	0.000	0.086	0.160
Target type: Self	-1.583	0.032	-49.012	0.000	-1.647	-1.520
Target type: Young	-1.016	0.026	-38.544	0.000	-1.067	-0.964
Graph Literacy (Centered)	-0.088	0.024	-3.657	0.000	-0.135	-0.041
COVID-19 Knowledge (Centered)	-0.009	0.016	-0.552	0.581	-0.041	0.023
Health Risk Factors (Centered)	0.059	0.032	1.858	0.063	-0.003	0.121
Contracted COVID-19 (Centered)	0.049	0.040	1.233	0.218	-0.029	0.128
Tested for COVID-19 (Centered)	0.074	0.028	2.639	0.008	0.019	0.128
Age (Centered)	-0.006	0.002	-2.362	0.018	-0.011	-0.001
Gender (Centered)	-0.083	0.026	-3.164	0.002	-0.135	-0.032
Education (Centered)	0.003	0.016	0.169	0.866	-0.029	0.034
**Time point *Visualization: No Forecast**	**-0.216**	**0.060**	**-3.598**	**0.000**	**-0.334**	**-0.098**
**Time point *Visualization: CI 50**	**-0.175**	**0.060**	**-2.918**	**0.004**	**-0.292**	**-0.057**
**Time point *Visualization: Gradient**	**-0.180**	**0.060**	**-3.016**	**0.003**	**-0.297**	**-0.063**
**Time point *Visualization: Gradient+Mean**	**-0.199**	**0.060**	**-3.337**	**0.001**	**-0.316**	**-0.082**
**Time point *Visualization: CI95 No Mean**	**-0.222**	**0.060**	**-3.709**	**0.000**	**-0.339**	**-0.105**
Time point * Y-axis	-0.013	0.035	-0.366	0.715	-0.080	0.055
Visualization: No Forecast *Y-axis	-0.022	0.181	-0.122	0.903	-0.376	0.332
Visualization: CI 50 *Y-axis	0.007	0.180	0.039	0.969	-0.346	0.360
Visualization: Gradient *Y-axis	-0.276	0.179	-1.541	0.123	-0.627	0.075
Visualization: Gradient+Mean *Y-axis	-0.174	0.180	-0.971	0.332	-0.527	0.178
Visualization: CI95 No Mean * Y-axis	0.043	0.180	0.241	0.810	-0.309	0.396

Effect size for the model:

```
##            R2m       R2c
## [1,] 0.1832356 0.5046165
```

4.2.5.1 Mean risk judgments for each condition

Table showing summary statics for each condition

Visualization	Y-axis	Time-point	N	Mean Judgment	sd	se	ci
6 Models	Cumulative	Pre	900	3.774	2.105	0.070	0.138
6 Models	Cumulative	Post	900	4.154	2.004	0.067	0.131
6 Models	Incident	Pre	900	3.941	1.974	0.066	0.129
6 Models	Incident	Post	900	4.399	1.821	0.061	0.119
CI 50	Cumulative	Pre	900	3.870	1.992	0.066	0.130
CI 50	Cumulative	Post	900	4.143	1.871	0.062	0.122
CI 50	Incident	Pre	900	4.037	2.142	0.071	0.140
CI 50	Incident	Post	900	4.291	2.055	0.069	0.134
CI 95 No Mean	Cumulative	Pre	900	3.657	1.985	0.066	0.130
CI 95 No Mean	Cumulative	Post	900	3.858	1.928	0.064	0.126
CI 95 No Mean	Incident	Pre	900	3.916	2.033	0.068	0.133
CI 95 No Mean	Incident	Post	900	4.110	1.957	0.065	0.128
Gradient	Cumulative	Pre	900	3.929	2.124	0.071	0.139
Gradient	Cumulative	Post	900	4.152	1.982	0.066	0.130
Gradient	Incident	Pre	900	3.809	1.969	0.066	0.129
Gradient	Incident	Post	900	4.061	1.901	0.063	0.124
Gradient+ Mean	Cumulative	Pre	900	3.787	2.069	0.069	0.135
Gradient+ Mean	Cumulative	Post	900	4.098	2.000	0.067	0.131
Gradient+ Mean	Incident	Pre	900	3.750	1.978	0.066	0.129
Gradient+ Mean	Incident	Post	900	3.907	1.917	0.064	0.125
No Forecast	Cumulative	Pre	900	3.753	2.124	0.071	0.139
No Forecast	Cumulative	Post	900	3.946	2.027	0.068	0.133
No Forecast	Incident	Pre	900	3.909	2.114	0.070	0.138
No Forecast	Incident	Post	900	4.138	2.023	0.067	0.132

4.2.6 Plot of primary task results

Results of the visualization comparison in Experiment 2 (ordered by size of the time point main effect), where pre-visualization risk judgments are colored gray and post-visualization judgments blue, for cumulative y-axis (top) and incident y-axis (bottom). Dashed lines show the mean pre- and post-visualization risk judgments for each y-axis group as a whole. Black bars show 95% confidence intervals around the mean (black dot) for each condition using the Cousineau-Morey method and the density plots were generated from this data.

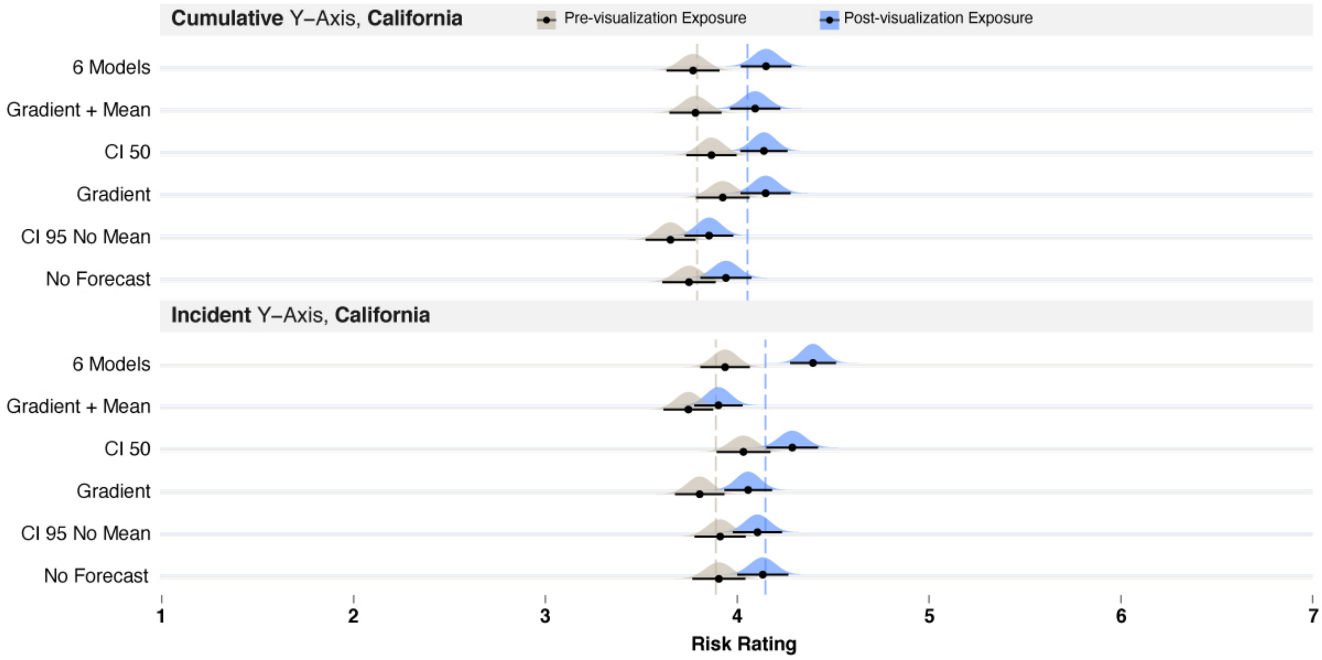

4.2.7 Interaction analysis: Time Point * 6 Models vs. all other visualizations

To break down these interactions, we ran separate models for each visualization. The models other than the 6 Models and No Forecast failed to converge, and we removed time point as a random slope from the model to support convergence (see example below).

Risk estimate (outcome) ∼
Time point x Y-axis (fixed-interaction) +
Time point (fixed-effect, categorical 2 levels, referent = pre-visualization exposure) +
Y-axis (fixed-effect, categorical 2 levels, referent = cumulative) +
Question type (fixed-effect, numeric, coded as -1, -.5, .5, 1) +
Target type (fixed-effect, categorical 3 levels, referent = self) +
Graph literacy centered centered (fixed-effect, numeric 0-4, centered on grand mean) +
COVID-19-knowlege centered (fixed-effect, numeric 1-13, centered on grand mean) +
COVID-19-health-risk centered (fixed-effect, numeric 1-20, centered on grand mean) +
Contracted COVID-19 centered (fixed-effect, dichotomous, yes = 1 no = -1) +
Tested for COVID-19 centered (fixed-effect, dichotomous, yes = 1 no = -1) +
Age centered (fixed-effect, numeric, centered on grand mean)+
Gender centered (fixed-effect, dichotomous, male = 1 female = -1) +
Education centered (fixed-effect, numeric 1-8, centered on grand mean) +
(Target type + Question type | Participant Id)

4.2.7.1 Model with participants in the 6 Models group These analysis revealed that each of the visualizations showed a significant increase in risk estimates (see 6-model Table below, highlighted in green). The 6-model visualization produced significantly larger increases in risk estimates (mean change of .43) compared to each of the other visualization techniques (mean change of .23 on average). The larger increase in risk estimates for 6 Models can be seen in Figure 4.2.6, were the visualization techniques are ordered by their average change.

	Estimate	Std..Error	t.value	p.z	2.5 %	97.5 %
(Intercept)	4.601	0.123	37.492	0.000	4.360	4.841
**Time point**	**0.380**	**0.054**	**7.034**	**0.000**	**0.274**	**0.486**
Y-axis	0.225	0.130	1.732	0.083	-0.030	0.479
Question type	0.143	0.048	2.973	0.003	0.049	0.238
Target type: Self	-1.517	0.085	-17.916	0.000	-1.683	-1.351
Target type: Young	-1.003	0.063	-15.838	0.000	-1.127	-0.878
Graph Literacy (Centered)	-0.086	0.062	-1.384	0.166	-0.208	0.036
COVID-19 Knowledge (Centered)	-0.109	0.040	-2.725	0.006	-0.188	-0.031
Health Risk Factors (Centered)	0.001	0.084	0.016	0.987	-0.164	0.166
Contracted COVID-19 (Centered)	0.019	0.094	0.204	0.838	-0.164	0.203
Tested for COVID-19 (Centered)	0.069	0.066	1.045	0.296	-0.060	0.198
Age (Centered)	0.003	0.007	0.418	0.676	-0.011	0.017
Gender (Centered)	-0.208	0.063	-3.309	0.001	-0.332	-0.085
Education (Centered)	0.002	0.044	0.036	0.971	-0.085	0.089
Time point *Y-axis	0.085	0.077	1.110	0.267	-0.065	0.235

4.2.7.2 Model with participants in the No Forecast group These analysis revealed a significant increase in risk estimates for No Forecast (see Table below, highlighted in green).

	Estimate	Std..Error	t.value	p.z	2.5 %	97.5 %
(Intercept)	4.812	0.121	39.899	0.000	4.576	5.048
**Time point**	**0.190**	**0.059**	**3.226**	**0.001**	**0.075**	**0.306**
Y-axis	0.165	0.132	1.252	0.210	-0.093	0.423
Question type	0.129	0.046	2.823	0.005	0.039	0.219
Target type: Self	-1.536	0.077	-20.019	0.000	-1.687	-1.386
Target type: Young	-1.033	0.070	-14.825	0.000	-1.169	-0.896
Graph Literacy (Centered)	-0.076	0.056	-1.348	0.178	-0.185	0.034
COVID-19 Knowledge (Centered)	-0.026	0.036	-0.716	0.474	-0.097	0.045
Health Risk Factors (Centered)	0.087	0.083	1.047	0.295	-0.076	0.251
Contracted COVID-19 (Centered)	0.264	0.095	2.783	0.005	0.078	0.450
Tested for COVID-19 (Centered)	0.077	0.064	1.202	0.229	-0.049	0.203
Age (Centered)	-0.003	0.007	-0.436	0.663	-0.016	0.010
Gender (Centered)	-0.033	0.065	-0.515	0.606	-0.160	0.094
Education (Centered)	0.014	0.038	0.363	0.717	-0.061	0.089
Time point *Y-axis	0.029	0.084	0.346	0.729	-0.136	0.194

4.2.7.3 Model with participants in the CI50 group These analysis revealed a significant increase in risk estimates for CI50 (see Table below, highlighted in green).

	Estimate	Std..Error	t.value	p.z	2.5 %	97.5 %
(Intercept)	4.809	0.120	40.160	0.000	4.574	5.044
**Time point**	**0.267**	**0.055**	**4.861**	**0.000**	**0.159**	**0.374**
Y-axis	0.220	0.131	1.676	0.094	-0.037	0.478
Question type	0.121	0.048	2.521	0.012	0.027	0.216
Target type: Self	-1.641	0.080	-20.496	0.000	-1.798	-1.484
Target type: Young	-1.020	0.073	-13.994	0.000	-1.162	-0.877
Graph Literacy (Centered)	-0.380	0.065	-5.867	0.000	-0.508	-0.253
COVID-19 Knowledge (Centered)	0.101	0.041	2.466	0.014	0.021	0.181
Health Risk Factors (Centered)	-0.085	0.075	-1.137	0.256	-0.233	0.062
Contracted COVID-19 (Centered)	0.101	0.094	1.078	0.281	-0.083	0.286
Tested for COVID-19 (Centered)	0.165	0.065	2.528	0.011	0.037	0.293
Age (Centered)	-0.009	0.006	-1.588	0.112	-0.021	0.002
Gender (Centered)	-0.097	0.063	-1.523	0.128	-0.221	0.028
Education (Centered)	-0.018	0.038	-0.467	0.641	-0.092	0.057
Time point *Y-axis	-0.039	0.078	-0.495	0.620	-0.191	0.114

4.2.7.4 Model with participants in the CI95 No Mean group These analysis revealed a significant increase in risk estimates for CI95 No Mean (see Table below, highlighted in green).

	Estimate	Std..Error	t.value	p.z	2.5 %	97.5 %
(Intercept)	4.481	0.120	37.315	0.000	4.246	4.717
**Time point**	**0.201**	**0.055**	**3.650**	**0.000**	**0.093**	**0.309**
Y-axis	0.193	0.140	1.379	0.168	-0.081	0.466
Question type	0.112	0.043	2.577	0.010	0.027	0.196
Target type: Self	-1.540	0.076	-20.304	0.000	-1.689	-1.391
Target type: Young	-1.006	0.065	-15.565	0.000	-1.133	-0.879
Graph Literacy (Centered)	0.076	0.059	1.289	0.197	-0.040	0.192
COVID-19 Knowledge (Centered)	-0.036	0.042	-0.860	0.390	-0.120	0.047
Health Risk Factors (Centered)	0.110	0.075	1.458	0.145	-0.038	0.257
Contracted COVID-19 (Centered)	-0.068	0.093	-0.731	0.465	-0.250	0.114
Tested for COVID-19 (Centered)	0.119	0.073	1.630	0.103	-0.024	0.261
Age (Centered)	0.003	0.006	0.520	0.603	-0.008	0.014
Gender (Centered)	-0.112	0.067	-1.655	0.098	-0.244	0.021
Education (Centered)	-0.088	0.038	-2.311	0.021	-0.163	-0.013
Time point *Y-axis	-0.001	0.079	-0.011	0.991	-0.155	0.153

4.2.7.5 Model with participants in the Gradient group

These analysis revealed a significant increase in risk estimates for Gradient (see Table below, highlighted in green).

	Estimate	Std..Error	t.value	p.z	2.5 %	97.5 %
(Intercept)	4.864	0.124	39.088	0.000	4.620	5.108
**Time point**	**0.226**	**0.054**	**4.157**	**0.000**	**0.120**	**0.333**
Y-axis	-0.105	0.128	-0.819	0.413	-0.356	0.146
Question type	0.156	0.043	3.651	0.000	0.072	0.240
Target type: Self	-1.647	0.080	-20.511	0.000	-1.804	-1.489
Target type: Young	-1.061	0.058	-18.275	0.000	-1.175	-0.947
Graph Literacy (Centered)	-0.087	0.052	-1.668	0.095	-0.190	0.015
COVID-19 Knowledge (Centered)	-0.049	0.048	-1.026	0.305	-0.143	0.045
Health Risk Factors (Centered)	0.010	0.069	0.151	0.880	-0.124	0.145
Contracted COVID-19 (Centered)	-0.010	0.097	-0.099	0.921	-0.199	0.180
Tested for COVID-19 (Centered)	0.099	0.067	1.477	0.140	-0.032	0.230
Age (Centered)	0.000	0.006	-0.065	0.948	-0.013	0.012
Gender (Centered)	-0.144	0.062	-2.338	0.019	-0.265	-0.023
Education (Centered)	-0.007	0.036	-0.207	0.836	-0.078	0.063
Time point *Y-axis	0.026	0.077	0.337	0.736	-0.125	0.176

4.2.7.6 Model with participants in the Gradient + Mean group

These analysis revealed a significant increase in risk estimates for Gradient + Mean (see Table below, highlighted in green).

	Estimate	Std..Error	t.value	p.z	2.5 %	97.5 %
(Intercept)	4.703	0.122	38.629	0.000	4.465	4.942
**Time point**	**0.306**	**0.055**	**5.611**	**0.000**	**0.199**	**0.413**
Y-axis	-0.023	0.132	-0.177	0.860	-0.283	0.236
Question type	0.076	0.049	1.541	0.123	-0.021	0.172
Target type: Self	-1.617	0.076	-21.343	0.000	-1.766	-1.469
Target type: Young	-0.972	0.057	-16.909	0.000	-1.085	-0.859
Graph Literacy (Centered)	0.012	0.056	0.218	0.828	-0.098	0.123
COVID-19 Knowledge (Centered)	0.024	0.032	0.729	0.466	-0.040	0.087
Health Risk Factors (Centered)	0.159	0.080	1.972	0.049	0.001	0.316
Contracted COVID-19 (Centered)	0.003	0.111	0.030	0.976	-0.215	0.221
Tested for COVID-19 (Centered)	0.056	0.073	0.769	0.442	-0.087	0.200
Age (Centered)	-0.016	0.005	-3.151	0.002	-0.026	-0.006
Gender (Centered)	0.060	0.064	0.937	0.349	-0.065	0.185
Education (Centered)	0.040	0.038	1.036	0.300	-0.035	0.115
Time point *Y-axis	-0.160	0.077	-2.071	0.038	-0.311	-0.009

5 Data and code availability

This report was generated in R-Markdown, and as such, it contains the complete analysis code. The R-markdown script and data are freely available on the Open Science Framework: https://osf.io/hfvqc/?view_only=7a29b9a097f5410696bfe4db7c0a99ba

References:

Azlan, Arina Anis, Mohammad Rezal Hamzah, Tham Jen Sern, Suffian Hadi Ayub, and Emma Mohamad. 2020. "Public Knowledge, Attitudes and Practices Towards COVID-19: A Cross-Sectional Study in Malaysia." *Plos One* 15 (5): e0233668.

Okan, Yasmina, Eva Janssen, Mirta Galesic, and Erika A Waters. 2019. "Using the Short Graph Literacy Scale to Predict Precursors of Health Behavior Change." *Medical Decision Making* 39 (3): 183–95.
